# Limited Benefits of Oyster Aquaculture on Water Clarity in Two Rhode Island Salt Ponds

**DOI:** 10.3390/coasts6010006

**Published:** 2026-02-16

**Authors:** Suzanne G. Ayvazian, Donald Cobb, Cathleen Wigand, Kenneth Miller, Natalie Schafer, Alexandra Beardwood, Sara Miller, Nia Bartolucci

**Affiliations:** 1 U.S. Environmental Protection Agency, Atlantic Coastal Environmental Science Division, 27 Tarzwell Drive, Narragansett, RI 02882, USA; 2 General Dynamics Information Technology, 3170 Fairview Park Drive, Falls Church, VA 22042, USA; 3 ORAU Student Services Contractor, U.S. Environmental Protection Agency, Atlantic Coastal Environmental Science Division, 27 Tarzwell Drive, Narragansett, RI 02882, USA; 4 Department of Earth and Environment, Boston University, Boston, MA 02215, USA

**Keywords:** oysters, aquaculture, water clarity, phytoplankton productivity

## Abstract

Shellfish restoration and aquaculture are considered as innovative methods to mitigate in-water nutrients in coastal waters. Water quality was examined at two oyster aquaculture farms in Potter (2021–2023) and Pt Judith (2023) Ponds in southern Rhode Island, USA. Twice monthly, on a flooding tide, upstream and downstream positions were established and water quality measures were made using fluorometry sondes and laboratory analysis. Significant differences in chlorophyll *a*, turbidity, and nutrient concentrations between upstream and downstream positions were identified; however, the differences were not consistently greater upstream or downstream. Percent Chl *a* removed varied from −74% to 64% at Potter Pond among years and −51% to 29% at Point Judith Pond, indicating a deficit or increase in Chl *a* concentration downstream as compared to upstream over each sampling period. Chlorophyll *a* measured inside oyster bags was higher compared to the upstream position at Potter Pond, and results from the upstream, downstream, and within farm productivity experiment in both ponds suggest oyster byproducts may facilitate localized and seasonal phytoplankton production. Natural resource managers should consider that while oyster farms in coastal ponds can provide local water clarity through removal of phytoplankton, benefits may be site specific and seasonal.

## 1. Introduction

Over the past two decades, commercial shellfish aquaculture, restorative aquaculture, and shellfish restoration, particularly using the Eastern oyster, *Crassostrea virginica* (Gmelin, 1791), have gained considerable popularity within the United States and internationally, as innovative and credible techniques for in-water nutrient remediation in estuaries and coastal waters experiencing nutrient over-enrichment and habitat destruction [1,2]. Coastal development has continued to urbanize natural landscapes and alter hydrology and further increase non-point source nutrient additions to coastal areas and watersheds [3]. It has long been recognized that nitrogen is one of the primary limiting nutrients for phytoplankton growth in coastal areas. Excessive nitrogen entering coastal water bodies from the watershed and airshed fuel phytoplankton growth leading to eutrophication, hypoxic events which negatively impact shallow water fish and invertebrate populations, and subsequent habitat loss of seagrasses due to shading [4–6]. Therefore, in addition to traditional engineered water management strategies, such as tertiary upgrades to wastewater treatment facilities, and advanced septic systems, incorporating oyster mediated nitrogen removal is an important in-water nutrient reduction strategy.

Bioextraction of oysters through harvest has been approved as a best management practice for the removal of nutrients in Chesapeake Bay to meet mandated nutrient reductions [7]. Oyster aquaculturalists can receive compensation for their harvest through the Maryland Nutrient Trading Program [8,9]. In Massachusetts, the Cape Cod Commission 208 plan was developed in response to total maximum daily loads placed on many Cape Cod estuaries and coastal waterbodies for nitrogen over-enrichment [10]. Many municipalities have developed their own oyster enhancement programs, including shellfish restoration and aquaculture, as innovative and alternative strategies to increase in-water nitrogen removal capacity [11,12]. In addition to their economic benefits, the expansion of commercial oyster aquaculture and restorative aquaculture farms in Southern New England have the potential to be utilized for nutrient reduction in future management strategies

Oysters and other suspension-feeding bivalve mollusks facilitate reductions in turbidity and enhance water clarity in the water column through the removal of suspended particulate and phytoplankton biomass (seston), through their filtration activities [13,14]. Controlled laboratory studies on multiple mollusk species have established clearance rates; however, variability has been reported when translating these values to in situ measures [15,16]. Watershed inputs, phytoplankton composition, water temperature, salinity, and oyster soft tissue biomass are factors influencing the magnitude of clearance rates [17,18]. In turn, bivalve consumption of seston removes nitrogen from the water column which is assimilated into the soft tissue biomass and shell for growth resulting in improved water clarity [19,20]. Using chlorophyll a (Chl *a*) concentration as a proxy for phytoplankton, in situ measures at oyster reefs and aquaculture facilities have demonstrated depletion of Chl *a* concentration at a position downstream of the tidal movement over the oysters suggesting phytoplankton consumption. Quantifying the magnitude of the seston removed has yielded variable results, precluding a consistent and generalizable pattern in Chl *a* flux at natural or restored oyster reefs [21–25] or oyster aquaculture farms [26,27].

The disparate results from these studies may be explained, in part, by the metabolic waste products resulting from oyster filtration activity which includes undigested particles as mucus bound pseudofeces, digested particles as feces, and ammonia and urea. The fate of accumulated biodeposits transferred onto the sediment include burial for long term storage, dispersal downstream of the aquaculture facility, and degradation by bacteria and release as dissolved nitrogen to the overlying water column [26]. The ammonia waste in the water column can stimulate nitrification and the growth of phytoplankton [28,29] and other algal species [30,31]. Through these processes, bivalves are credited with coupling the pelagic-benthic environs and are considered an important component of the ecology of shallow coastal systems.

Worldwide studies have demonstrated benefits of shellfish aquaculture to water clarity [32–35] but few studies have appraised the performance of oyster farms in shallow, vegetated coastal ponds to provide improved water clarity in the Northeastern region of the USA [36]. Quantifying the benefit of water quality improvement is of particular importance in coastal Northeast USA as bivalve removal of excessive seston improves water clarity and increases light reaching the benthos, potentially providing conditions for the regrowth of decimated eelgrass, *Zostera marina*, populations [37]. Eelgrass is another essential fish and invertebrate habitat and along with oysters, contributes to water clarity and, therefore, is an important species to conserve [38].

Importantly, natural resource managers are considering the use of shellfish aquaculture and restoration as remediation tools for in-water nutrient mitigation; however, there are few studies in this region on which to base management plans. Therefore, the objectives of this study were to (1) quantify the differences in Chl *a* concentrations (as a proxy for phytoplankton), turbidity, nutrients, and environmental parameters between the upstream and downstream sampling positions at two oyster aquaculture farms in Rhode Island coastal ponds; and (2) quantify the Chl *a* concentrations within the Potter Pond aquaculture farm bags (2021) and compare with the Chl *a* concentrations from the incoming water (2021). We anticipated that Chl *a* and turbidity values recorded at the downstream sampling position would be significantly lower than that recorded upstream at both farms and for each sampling date. Inconsistent findings might suggest the oyster waste products contribute to the regeneration of phytoplankton both within the farm bags as well as the overall farm. To further examine this second objective, a pilot light:dark bottle productivity experiment was conducted at the two aquaculture farms in 2024.

## 2. Material and Methods

### 2.1. Water Clarity Field and Laboratory Study

**Figure 1.**
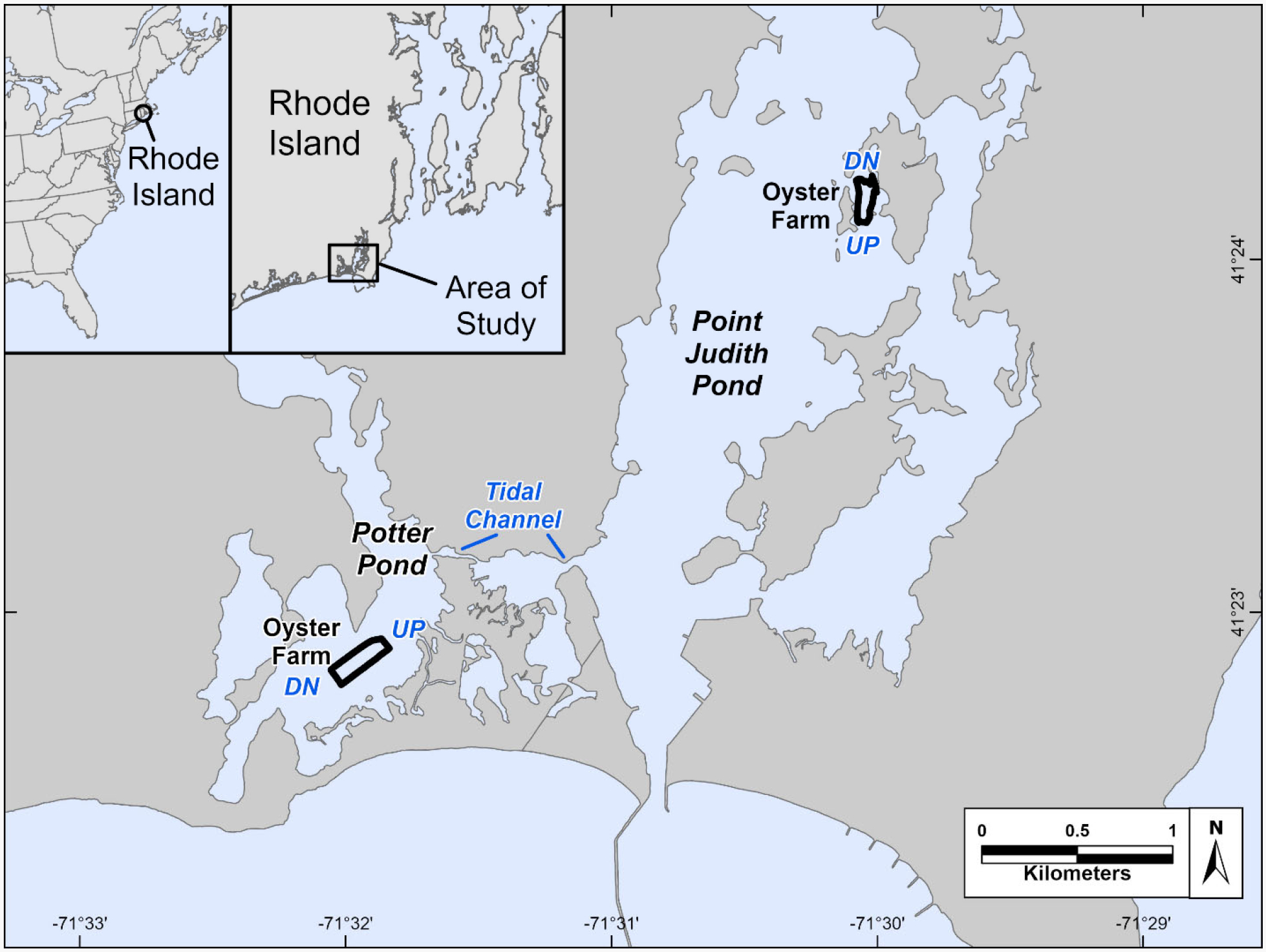
Location map of the commercial oyster aquaculture farms in Potter Pond and Pt Judith Pond, Rhode Island, RI, USA. Inset 1 is the Atlantic seaboard of the USA with the state of RI highlighted. Inset 2 is the state of RI showing the area of study. The detailed map shows Pt Judith and Potter Ponds, the tidal channel connecting the two ponds, the inlet to Block Island Sound on the southern border of Pt Judith Pond, and the location of the two oyster farms. Sampling was undertaken during a flooding tide and the upstream (UP) and downstream (DN) sampling positions are indicated.

This study was conducted at two commercial oyster aquaculture farms located in the subtidal waters of two vegetated coastal salt ponds in Southern Rhode Island, IR, USA. Matunuck Oyster Farm is a 2.8 hectare farm located in Potter Pond (41.394 N, −71.53 W) (henceforth referred to as Potter Pond site). This farm is positioned north–east to south–west approximately parallel to the barrier beach. There is no freshwater stream input into this well mixed coastal pond. Average water depth was 0.67 m and average water flow during the flood tide was 139 m h^−1^ across sampling years 2021–2023. The oyster culture method is off bottom with oysters contained in mesh bags. Jonathan Island Farm is a 2.4-hectare farm located in Point Judith Pond, RI (41.399 N, −71.505 W) (henceforth referred to as Pt Judith Pond site). The farm is oriented north–south, along the same directional axis as the pond and is located approximately (~3 km) midway between the northern boundary of the pond and the oceanic opening. Freshwater enters the northern end of the pond via two small streams. Average values of water depth and water flow rate at the site during the flood tide were 0.85 m and 188 m h^−1^, respectively. The diurnal tidal exchange and flow rate ensured a well-mixed water column at the farm site. This farm uses suspended culture with attached mesh oyster bags. The Potter Pond (131.5 hectares)–Point Judith Pond (607.0 hectares) complex is a microtidal wave-dominated coastal environment [39]. The two ponds are connected by a tidal channel approximately 800 m long [40] (Figure 1). These two farms were chosen for study from the more than 80 oyster aquaculture operations in Rhode Island waters for the large acreage of the farm and the accessibility to the sites. In addition, each farm used different culture practices which allowed us to examine the robustness of the benefits of oyster farms to water quality. Sampling for water quality at Potter Pond was conducted twice monthly from 19 May to 27 September 2021, inclusive (*n* = 10 visits), 7 June to 2 November 2022, inclusive (*n* = 10 visits), and 13 April to 5 October 2023, inclusive (*n* = 12 visits), and at Pt Judith Pond from 15 May to 10 October 2023, inclusive (*n* = 9 visits).

Field sampling occurred approximately two hours after the initiation of a flooding tide and lasted for two hours. This period was chosen specifically as measurable water flow across the oyster farm brought a fresh supply of seston from oceanic waters. A Marsh McBurnie electromagnetic flowmeter (Model 2000, Marsh McBurnie Inc. Frederick, MD, USA) was used to measure water flow speed and direction to determine the upstream and downstream designations at the boundaries of the aquaculture farm. A compass was used to determine water flow and wind directions. A Secchi disk was used to measure the depth of water transparency (m). Water depth (m) was measured and recorded. Wind speed estimates were derived from local observations applied to the Beaufort wind speed chart. Hydrolab sondes (OTT HydroMet Brand, Sterling, VA, USA) were positioned strategically ≥ 2 m upstream and downstream of the aquaculture site and set to record chlorophyll *a* (Chl *a*; μg L^−1^), turbidity (mg L^−1^), water temperature (°C), salinity (PSU), dissolved oxygen (DO; mg L^−1^) every 15 min for 2 h (*n* = 9 samples/visit). Hydrolab sondes were calibrated according to manufacturer’s recommendation prior to each sampling event. The sondes were attached to metal gardening stakes and positioned mid-water column, at the same average depth as the aquaculture gear.

In addition to the Hydrolab measurements, duplicate water samples were collected manually and from same water depth and area as the sonde, to measure Chl *a* every 15 min for 2 h to coincide with the HydroLab recordings at the upstream and downstream sites. Chlorophyll *a* was assessed by collecting 60 mL of raw seawater in an acid-stripped 60 mL syringe and passing it through a Swinnex filter tip (Merck KGaA, Darmstadt, Germany) containing a glass microfiber filter (Whatman GF/F 0.7 micron, Cytiva, Cardiff, UK). The GF/F filter was removed from the Swinnex filter tip, wrapped in aluminum foil on site, placed in a labelled plastic bag on ice in a cooler and returned to the laboratory. Filters were stored in the freezer prior to an acetone extraction and analyzed within two weeks of collection on the Turner Trilogy Fluorometer (San Jose, CA, USA). In 2021 only, additional water samples were collected from outside of the farm area and from within the aquaculture mesh bags holding oysters within 3 m of the upstream position at Potter Pond to examine the Chl *a* concentrations inside the mesh bags versus the Chl *a* of the incoming water.

The seston removed (as Chl *a*) by the oysters on the farm (and potentially other filter feeders associated with the aquaculture gear which were not quantified during this study) for each sampling date followed the formulation of similar studies [24,41] and was calculated as,

(1) $$\%\;\text{Seston}(\text{as}\;\text{Chl}\;a)\text{removed}=({\text{C}}_{1}-{\text{C}}_{2})/{\text{C}}_{1}\times 100$$

where: C_1_ = upstream Chl *a* values, and C_2_ = downstream Chl *a* values. Positive values signify a deficit in the percent seston downstream as a result of oyster feeding, while negative values indicate higher Chl *a* concentrations downstream.

Turbidity samples were collected in labelled acid-stripped 1 L brown plastic bottles at the beginning and end of the two-hour sampling period from the upstream and downstream sites, kept on ice in a cooler, and returned to the laboratory for processing. In the laboratory, water was passed through a clean pre-weighed GF/F filter positioned on top of a vacuum pump. The GF/F filter was placed in a drying oven at 60 °C for 24 h and reweighed. The difference in weight between the filter with dried particulate matter and the clean filter was the measure of turbidity.

At the initial (1st), middle (5th), and last (9th) sampling time, dissolved inorganic nutrient samples were collected by filtering 60 mL of water through a glass fiber filter (Whatman GF/F 0.7 micron) into duplicate labelled acid-washed, de-ionized water leached, polyethylene vials. Vials were kept in labelled plastic bags on ice in a cooler, returned to the laboratory, and placed in the freezer until analyzed. Subsequently, samples were analyzed on the Astoria Pacific Auto-Analyzer (Clackamas, OR, USA). Nutrient samples were analyzed for ammonia (NH_3_^+^), nitrite (NO_2_^−^), nitrate plus nitrite (NO_3_^−^ + NO_2_^−^; together NO_x_^−^), and phosphate (PO_4_^3–^) concentrations using an Astoria-Pacific Astoria 2 Analyzer. Nitrate plus nitrite and nitrite were measured by cadmium reduction using US EPA method 353.2 [42]. Ammonia and phosphate were measured via U S EPA methods 350.1 [43] and 365.2 [44], respectively. Detection limits for the methods provided by Astoria were 0.02 mg L^−1^ for NH_3_^+^, 0.01 mg L^−1^ for NO_2_^−^, 0.01 mg L^−1^ for NO_x_^−^, and 0.05 mg L^−1^ for PO_4_^3–^. The stock standards were from NSI Laboratory solutions and were measured against check standards from Ricca Chemical after every 15 samples. All chemical analyses were conducted at the US EPA Atlantic Coastal Environmental Sciences Division (ACESD) in Narragansett, RI, USA.

Separate multi-factor General Linear Models (GLMs) were executed for each year and water quality parameter. In each model, position (upstream, downstream), date, and a date by position interaction term was included. While position was the primary factor of interest in the study, including the sampling date in each model controlled for seasonal and other temporal effects that would otherwise inflate the model variability and mitigate the ability to measure the impact of the position. The interaction term evaluated whether any sampling position effects were consistent across all sampling dates within the year. Date-specific comparisons were evaluated using Bonferroni adjustment when statistically significant date by position interactions were found. Annual comparisons at Potter Pond (2021–2023) were performed using a multi-factor GLM, with year and position as factors, and with date included as a nested factor within year. When no year by locations interactions were revealed pairwise comparisons of years were performed using Tukey–Kramer tests, and when a significant year by location interaction was found, the Bonferroni adjustment was applied. For the year-specific and annual comparison models, all measurements were natural log-transformed other than dissolved oxygen, temperature, and salinity. A two-factor GLM was performed to compare Chl *a* concentrations from inside the oyster mesh bag, outside the farm area, and at the upstream position at the farm, with location and day as factors, as well as the location by day interaction term. The raw data were natural-log transformed. Pairwise comparisons were conducted using the Tukey–Kramer test. The level of statistical significance was set at *p* < 0.05. Error measures are reported as ± 1 standard deviation (SD). Associations between Chl *a* and measured environmental variables were analyzed using the Spearman rank correlations with Chl *a*, turbidity, and nutrients log-transformed prior to analyses [45].

### 2.2. Productivity Experiment

The oxygen light:dark bottle method of quantifying the phytoplankton production around and within the aquaculture farms was employed during the 2024 oyster growing season [46,47]. Recognizing the potential for bottle effects (variation in light intensity throughout the day, increases in bacterial production, nutrient limitations, and presence of microzooplankton), short in situ incubation times were chosen [47]. On one occasion during the spring, summer, and autumn 2024, the light:dark bottle field experiment was conducted at each of the two aquaculture farms. Water was collected from upstream, downstream, and within farm positions into acid-washed, labelled light and dark bottles. Duplicate light bottles were filled from the three positions to measure the initial dissolved oxygen (DO, mg L^−1^) and water temperature (°C) using a YSI 58 DO meter with a YSI 5905 BOD bottle probe (YSI, Inc. Yellow Springs, OH, USA). Triplicate light and dark bottles were filled for each of the three positions (9 light and 9 dark bottles). After the two-hour incubation, DO and temperature were measured in the light and dark bottles from all positions and recorded. At the time of water collection, water temperature, salinity, DO, and pH were measured using a YSI Professional Plus water 198 sampler (YSI, Yellow Springs, OH, USA) and recorded. Water samples were collected from the upstream, downstream, and within farm positions for Chl *a* and nutrient concentrations, with laboratory analyses as described previously. The following equations were used to calculate net daily productivity.

(2) $$\text{Net}\;{\text{O}}_{2}\;\text{Production}(\text{mg}\;{\text{L}}^{-1})=\text{Initial}\;\text{DO}\;\text{from}\;\text{light}\;\text{bottle-final}\;\text{DO}\;\text{from}\;\text{light}\;\text{bottle},$$

(3) $$\text{Net}\;\text{Carbon}\;\text{Production}\;(\text{mg}\;\text{C}\;{\text{L}}^{-1})=\text{Net}\;{\text{O}}_{2}\;\text{production}\times 0.375(\text{conversion}\;\text{factor}\;\text{used}\;\text{for}\;\text{estimating}\;\text{C}\;\text{production}\;\text{from}\;\text{changes}\;\text{in}\;\text{dissolved}\;\text{oxygen}\;(0.375\;\text{mg}\;\text{C}\;\text{per}\;\text{mg}\;{\text{O}}_{2})),$$

(4) $$\text{Net}\;\text{Carbon}\;\text{Production}\;\text{Rate}\;(\text{mg}\;\text{C}\;{\text{L}}^{-1}{\text{h}}^{-1})=\text{Net}\;\text{C}\;\text{production}/\text{incubation}\;\text{time}\;(2\;\text{h}),$$

(5) $$\text{Net}\;\text{Daily}\;\text{Productivity}\;(\text{g}\;\text{C}\;{\text{m}}^{-3}{\text{day}}^{-1})=\text{Net}\;\text{C}\;\text{production}\;\text{rate}\times 24\;\text{h}.$$

Statistical analyses for daily productivity and nutrients were performed with a GLM, using untransformed productivity and natural log-transformed nutrient data. Chlorophyll *a* concentrations were evaluated similarly, but with analyses performed on the means calculated across the individual replicates. Pairwise comparisons were performed using Bonferroni adjustment when statistically significant interactions were present and using the Tukey–Kramer adjustment when interactions were not present. Associations between (natural log) Chl *a* concentrations, net daily productivity, and nutrient concentrations were evaluated with Pearson correlation coefficients [45].

## 3. Results

### 3.1. Potter Pond

Water temperatures followed expected seasonal patterns with the lowest water temperatures in either the spring or the fall and the warmest temperatures in the summer. The range of temperatures was 16.0–27.6 °C in 2021, 15.1–28.3 °C in 2022, and 13.4–30.4 °C in 2023 (Table 1). There were significant differences noted in 2021 and 2022 between the upstream and downstream water temperature values (2021 F = 11.07, *p* < 0.0001; 2022 F = 120.92, *p* < 0.0001); however, the sampling position differences were not consistent among years (Tables S1 and S2). In 2021, on seven sampling dates, water temperature recorded downstream was significantly greater than upstream, while in 2022, there were two sampling dates where downstream was greater than upstream and one occurrence with the reverse. Salinity values were indicative of estuarine conditions with an average of 29.1 ppt in 2021, 31.7 ppt in 2022, and 29.9 ppt in 2023 (Tables 1 and S2). Dissolved oxygen levels demonstrated significant date by position interactions for each year at Potter Pond oyster farm, although there was no definitive upstream or downstream or temporal pattern associated with these differences (Tables 1, S1 and S2). There were no annual differences in DO levels (Table 2). Water depth (m) measured at the upstream and downstream sampling positions ranged between 0.5 to 0.9 m. Secchi depth (m) was the same as water depth during each sampling.

**Figure 2.**
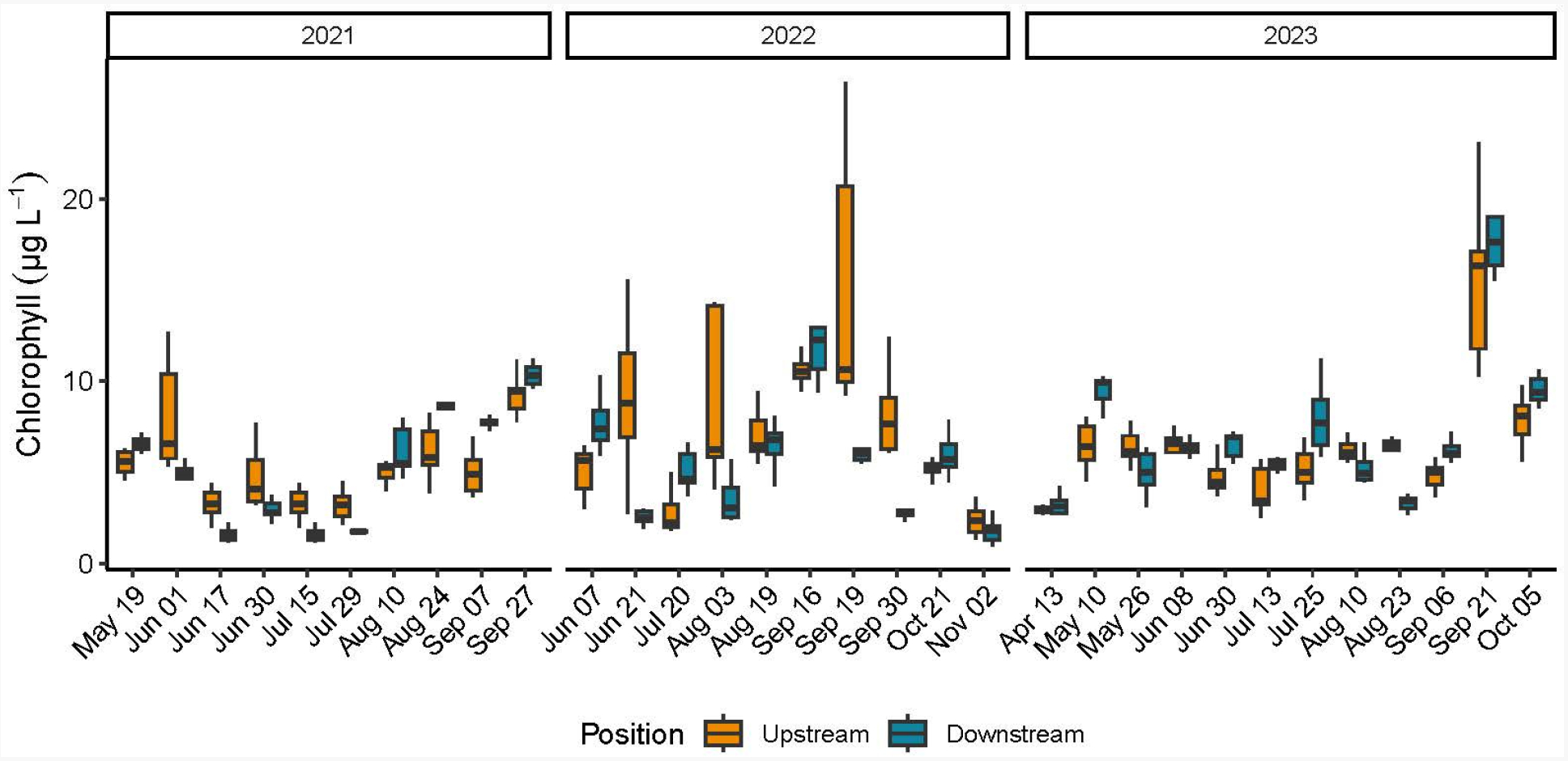
Box and whisker plot including the minimum value, first quartile, median, third quartile, and maximum value of chlorophyll *a* concentrations (μg L^−1^) determined using laboratory fluorometry from water samples collected upstream and downstream every 15 min over a two-hour sampling window during the oyster growing season at the Potter Pond commercial aquaculture farm between May 2021 and October 2023.

Chlorophyll *a* concentrations were evaluated with both the automated Hydrolab sondes and the hand collected filtered water samples analyzed with the Turner fluorometer. Unfortunately, multiple sonde failures in recording Chl *a* concentrations during sampling at Potter Pond oyster farm over three years rendered these data unreliable; therefore, sonde Chl *a* data are not reported. Overall, the fluorometer Chl *a* individual sample concentrations ranged between a minimum value of 0.01 μg L^−1^ (2023 upstream) and a maximum value of 36.98 μg L^−1^ (2022 upstream) (Tables 3 and S2). A significant relationship between position and date for Chl *a* concentrations was found for each year (2021 F = 19.15, *p* < 0.0001; 2022 F = 16.21, *p* < 0.0001; 2023 F = 3.41, *p* = 0.0003) (Table S1). When evaluated on a date-specific basis, positional differences were observed on 14 of the 32 dates (44%) across the three years. Of these 14 dates, the mean (*n* = 9) Chl *a* concentration at the upstream position was significantly > mean downstream Chl *a* concentration on 10 dates with the upstream values approximately twice as great as those measured downstream, and on four dates, the mean downstream Chl *a* concentration was significantly > upstream Chl *a* concentrations. The remaining dates (18 of the 32 dates) showed no significant position differences (Figure 2, Tables S1 and S2). The position differences did not follow a consistent seasonal or other temporal pattern for any of the years. Annual comparison of Chl *a* concentrations upstream and downstream among sampling dates did not demonstrate significant results (Table 2).

During the 10 sampling dates in 2021, there were nine dates where duplicate water samples were collected from outside the farm area (100 m away from the boundary of the farm) and five dates where duplicate water samples were drawn from inside the oyster bags closest to the incoming water to compare with the upstream Chl *a* concentrations. Chlorophyll *a* values from outside of the farm area ranged from 2.36 μg L^−1^–7.35 μg L^−1^ (average = 4.42 ± 1.82 μg L^−1^), the upstream position values ranged from 1.99 μg L^−1^–12.71 μg L^−1^ (average 5.383 ± 2.38 μg L^−1^), while the range of Chl *a* concentrations from inside the farm bags ranged from 1.99 μg L^−1^– 19.39 μg L^−1^ (average = 10.96 ± 6.66 μg L^−1^). There were significant main effects for the source of the water sample (F = 28.98, *p* < 0.0001) and date of the sampling (F = 17.97, *p* < 0.0001) with Chl *a* values inside of the oyster bag higher than either outside the farm area or from the upstream position at the farm.

The calculated % seston (Chl *a*) removed by the farm oysters showed considerable variability by sampling date; in 2021, the range of values was −49.34–51.24% (average 9.06%); in 2022, the range of values was −74.0–73.3% (average 14.3%); and in 2023, the range was −53.9–47.0% (average −14.6%) (Table S3).

**Figure 3.**
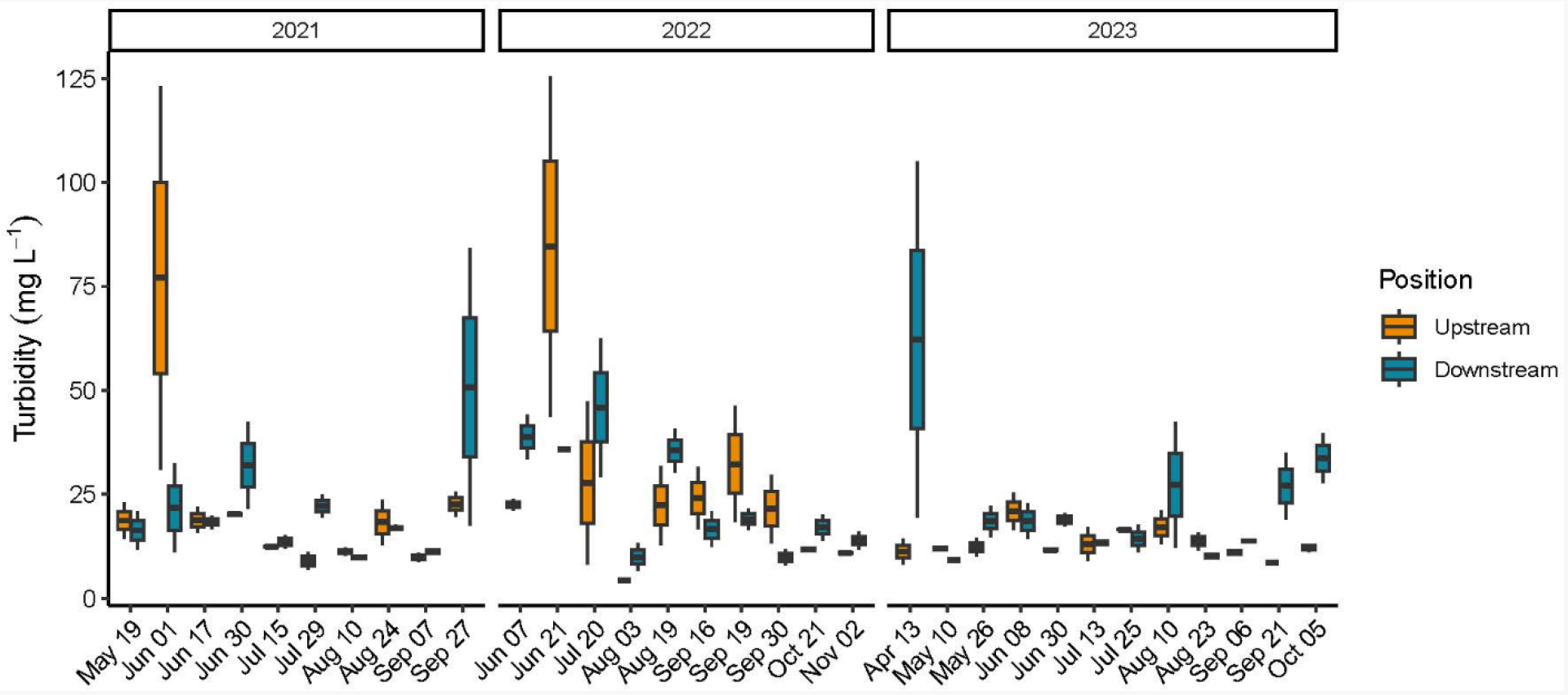
Box and whisker plot including the minimum value, first quartile, median, third quartile, and maximum value of turbidity concentrations (mg L^−1^) from water samples collected upstream and downstream at the start and end of the two-hour sampling window at the Potter Pond commercial aquaculture farm between May 2021 and October 2023.

Turbidity concentrations were evaluated with both the automated Hydrolab sondes and hand collected water samples which were processed in the laboratory. As with the Chl *a* sensor, there were multiple sonde failures for the turbidity sensor and these data are not reported. Turbidity values from the laboratory analysis of the water samples across the three years ranged between 4.29 mg L^−1−^ 125.5 mg L^−1^ with both values recorded during the 2022 field season at the upstream position (Tables 1 and S1). Surprisingly, no significant date by position interactions were reported for 2021 and 2022; however, the downstream position had consistently higher turbidity values than the upstream position in 2023 (F = 6.20, *p* = 0.0178) (Table S1). There were no annual differences in measured turbidity (Table 2, Figure 3).

**Figure 4.**
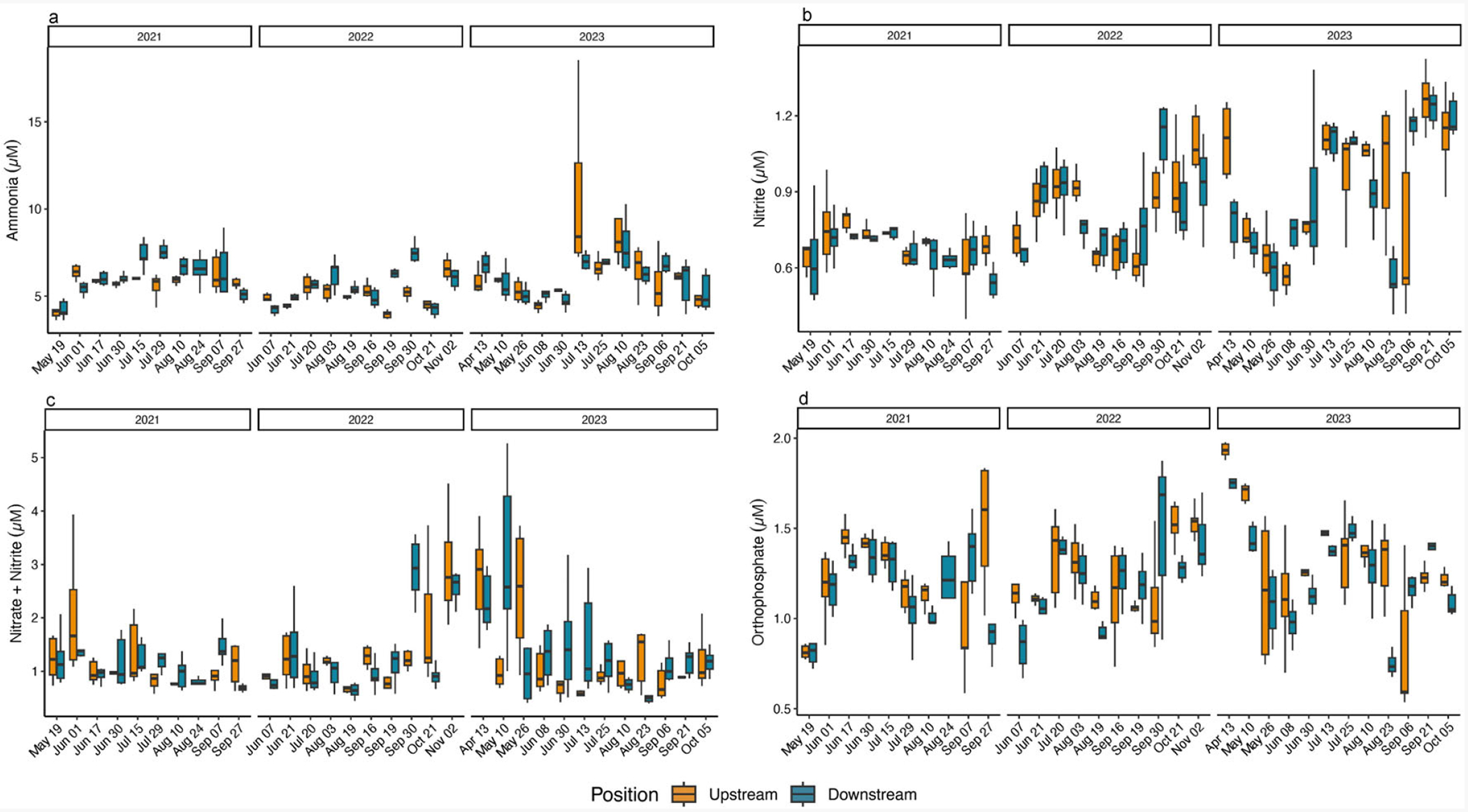
Box and whisker plot including the minimum value, first quartile, median, third quartile, and maximum value of (**a**). ammonia, (**b**). nitrite, (**c**). nitrate plus nitrite, and (**d**). orthophosphate concentrations (μM), respectively, from water samples collected upstream and downstream at the start, middle, and end of the two-hour sampling window at the Potter Pond commercial aquaculture farm between May 2021 and October 2023.

For all years, the average NH_3_^+^ concentration measured at the upstream position (annual means range from 5.10 (±0.81) μM–6.45 (±2.63) μM), was similar to the downstream position (annual means range from 5.53 (±1.12) μM–6.34 (±2.46) μM). In 2023, there was a doubling of the maximum value of upstream and downstream NH_3_^+^ concentration (Tables 4 and S2). Date by position interactions were observed for both 2021 and 2022 (2021 F = 4.38, *p* = 0.0002; 2022 F = 12.98, *p* < 0.0001) with NH_3_^+^ concentrations measured downstream > upstream on one sampling date in 2021, and on three sampling dates in 2022 (Table S1). Annual comparisons revealed an overall year difference with 2023 > 2022 (F = 4.36, *p* = 0.0229) (Table 2, Figure 4a).

The average NO_2_^−^ concentrations measured upstream (annual means range 0.70 (±0.10) μM–0.93 (±0.27 μM) was similar to that measured downstream (annual means range 0.66 (±0.11) μM–0.92 (±0.26) μM), with slightly increasing values noted between 2021 and 2023. There was no significant date by position interaction or significant differences between upstream and downstream positions in 2021; however, in 2022 there was a significant date by position interaction with downstream values > upstream values on 30 September (F = 3.16, *p* = 0.0021), and in 2023, a significant date by position interaction (F = 7.32, *p* < 0.0001) showed upstream values > downstream values on 23 August, and downstream values > upstream values on 8 June and 6 September (Figure 4b, Tables S1 and S2). The annual comparison showed a significant difference between years (F = 5.49, *p* = 0.0100) with 2023 NO_2_^−^ concentrations > 2021 NO_2_^−^ concentrations (Table 2) with the average NO_2_^−^ concentrations increasing among years at the upstream position by ~0.23 μM, and at the downstream position by ~0.26 μM (Table 4).

Upstream average nitrate plus nitrite (NO_x_^−^) concentrations were consistent between years (annual means range 1.28 (±0.84) μM–1.37 (±0.86) μM) and downstream average NO_x_^−^ concentrations were generally low among years, with slightly higher concentrations in 2022 and 2023 (downstream annual means range 1.14 (±0.52) μM–1.50 (±1.11) μM) (Tables 4 and S1). A significant date by position interaction was found in 2022 (F = 3.63, *p* = 0.0006), with average downstream values significantly > upstream values on 30 September, while upstream values > downstream values on 21 October. During 2023 sampling, the upstream values > downstream on 26 May and 23 August, and downstream values > upstream on 10 May (F = 4.29, *p* < 0.0001 for the date by position interaction) (Table S1). No annual differences in NO_x_^−^ values were observed at either sampling position (Table 2, Figure 4c).

While not statistically significant, there was a slight increase in the average PO_4_^3−^ upstream (annual means range 1.24 (±0.24) μM–1.32 (±0.34) μM) and downstream (annual means range 1.15 (±0.24) μM–1.24 (±0.29) μM) concentrations from 2021 to 2023 (Tables 4 and S1). Each year reported significant date by position interactions (2021 F = 4.45, *p* = 0.0001; 2022 F = 4.76, *p* < 0.0001; 2023 F = 6.21, *p* < 0.0001). Upstream PO_4_^3−^ values > downstream PO_4_^3−^ values on 27 September, and downstream PO_4_^3−^ values > upstream PO_4_^3−^ values on 7 September in 2021. The 2022 upstream PO_4_^3−^ values > downstream PO_4_^3−^ values on 7 June, while on 30 September the downstream values > upstream values. Similarly, in 2023, upstream values were significantly > downstream on 23 August, and downstream values > upstream on 6 September (Figure 4d, Table S1). Even with these occasional significant differences by date there were no overall annual differences (Table 2). Spearman rank correlations of Chl *a* and turbidity with the measured biophysical variables (temperature, salinity, DO, nutrients, wind speed and direction, current speed and direction, water depth, and Secchi depth) did not produce scientifically noteworthy associations across sampling dates and years.

One-time measures of seasonal net daily productivity collected at Potter Pond farm upstream, downstream, and within the oyster farm in spring (May), summer (July), and autumn (September) 2024 demonstrated a significant position by season interaction (F = 23.93, *p* < 0.0001). The summer productivity samples downstream > upstream and within farm positions, while the autumn productivity samples at the upstream position > downstream and within farm positions (Table 5). Net daily productivity measured at the three positions showed differences by season. In the upstream position, the autumn value > spring > summer, at the within farm position the spring value > summer and autumn values, and at the downstream position spring and summer productivity > autumn (Table 5).

Average Chl *a* concentrations across seasons and positions ranged between 5.29 (±0.31) μg L^−1^–10.48 (±1.06) μg L^−1^. A significant season by position interaction (F = 59.76, *p* < 0.0001) was demonstrated with Chl *a* concentrations in the downstream and within farm positions > upstream position during spring sampling. Chlorophyll *a* concentration in the downstream position > within farm position > upstream concentrations during summer. In autumn, the reverse result showed the upstream Chl *a* concentration > either downstream or within farm concentrations (Table 5).

Ammonia concentration did not exhibit a significant season by position interaction but had a notable overall season difference (F = 10.24, *p* = 0.0007) with summer (mean 7.00 ± 1.13 μM) and autumn (7.66 ± 2.18 μM) NH_3_^+^ concentrations > spring concentrations (5.29 ± 0.33 μM). Average NO_2_^−^ concentrations ranged between a mean of 3.30 (±1.15) μM across positions in the spring to a mean of 1.24 (±0.09) μM across positions in the autumn. Nutrient analyses of NO_2_^−^ demonstrated a number of significant position by season interactions (F = 3.29, *p* = 0.0343), specifically, the summer upstream NO_2_^−^ concentration > within farm NO_2_^−^ concentration. At the downstream position, spring NO_2_^−^ concentration > autumn NO_2_^−^ concentration, at the within farm position the NO_2_^−^ concentration in spring > either summer or autumn NO_2_^−^ concentration, and at the upstream position, the summer NO_2_^−^ concentration > autumn NO_2_^−^ concentration. Nitrite plus nitrate concentration showed a significant overall season difference (F = 4.94, *p* = 0.0169) with average spring values at all positions (4.83 ± 1.28 μM) > average autumn values at all positions (2.53 ± 1.40 μM). Ortho-phosphate concentrations demonstrated an overall season difference (F = 11.18, *p* = 0.0004) with the average summer (1.24 ± 0.22 μM) and fall (1.43 ± 0.21 μM) values at all positions > average spring (0.99 ± 0.12 μM) values at all positions (Table 5).

### 3.2. Pt. Judith Pond

There were nine sampling dates at the Jonathan Island Aquaculture Farm in Pt. Judith Pond between May 15 and October 10, 2023, inclusive. Water temperatures were lowest (17.5 °C) in October and highest (31.0 °C) in July (Tables 1 and S1). Salinity values ranged from 20.2 ppt at the upstream position in July to 32.4 ppt at the downstream position in June (Tables 1, S1 and S2). Dissolved oxygen sample concentrations ranged between a low value of 5.42 mg L^−1^ downstream to a high of 13.0 mg L^−1^ upstream (Tables 1, S1 and S2). Water depth values ranged between 0.6 m and 1.2 m with Secchi disk having the same measured values as water depth.

**Figure 5.**
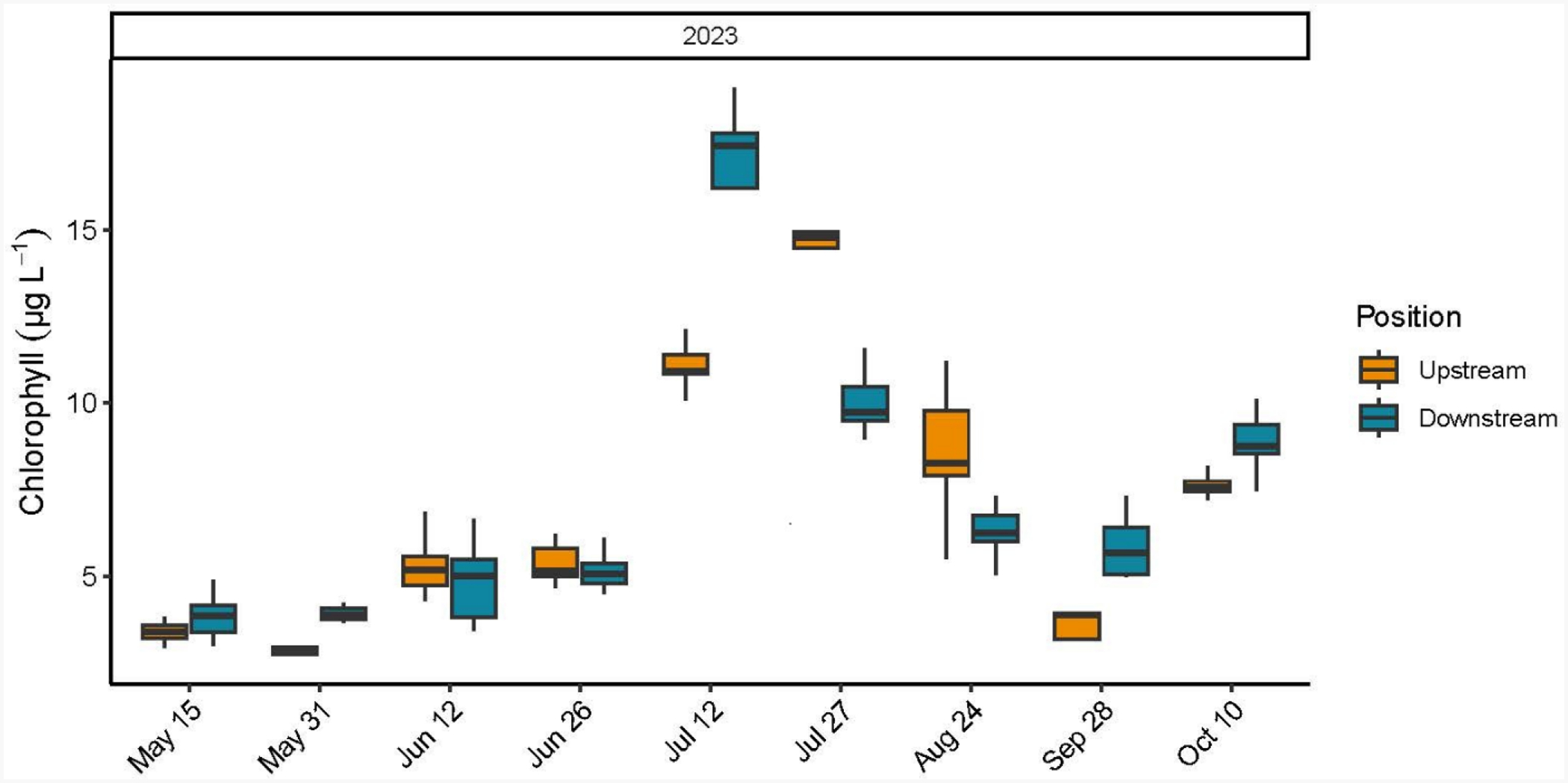
Box and whisker plot including the minimum value, first quartile, median, third quartile, and maximum value of chlorophyll *a* concentrations (μg L^−1^) determined using laboratory fluorometry from water samples collected upstream and downstream every 15 min over a two-hour sampling window during the oyster growing season at the Point Judith Pond commercial aquaculture farm between May and October 2023.

Chl *a* concentrations ranged between a minimum of 2.40 μg L^−1^ and a maximum of 23.67 μg L^−1^. A significant date by position interaction was determined (F = 12.96, *p* < 0.0001) where upstream Chl *a* concentrations > downstream Chl *a* concentrations on 27 July and 24 August, and downstream Chl *a* concentrations > upstream Chl *a* concentrations on 31 May, 12 July, and 28 September (Table 3 and Tables S1, Figure 5). The % seston removed by the farm oysters, across the nine sampling dates, ranged between −50.5% to 30.0% (average −5.7%) (Table S3).

**Figure 6.**
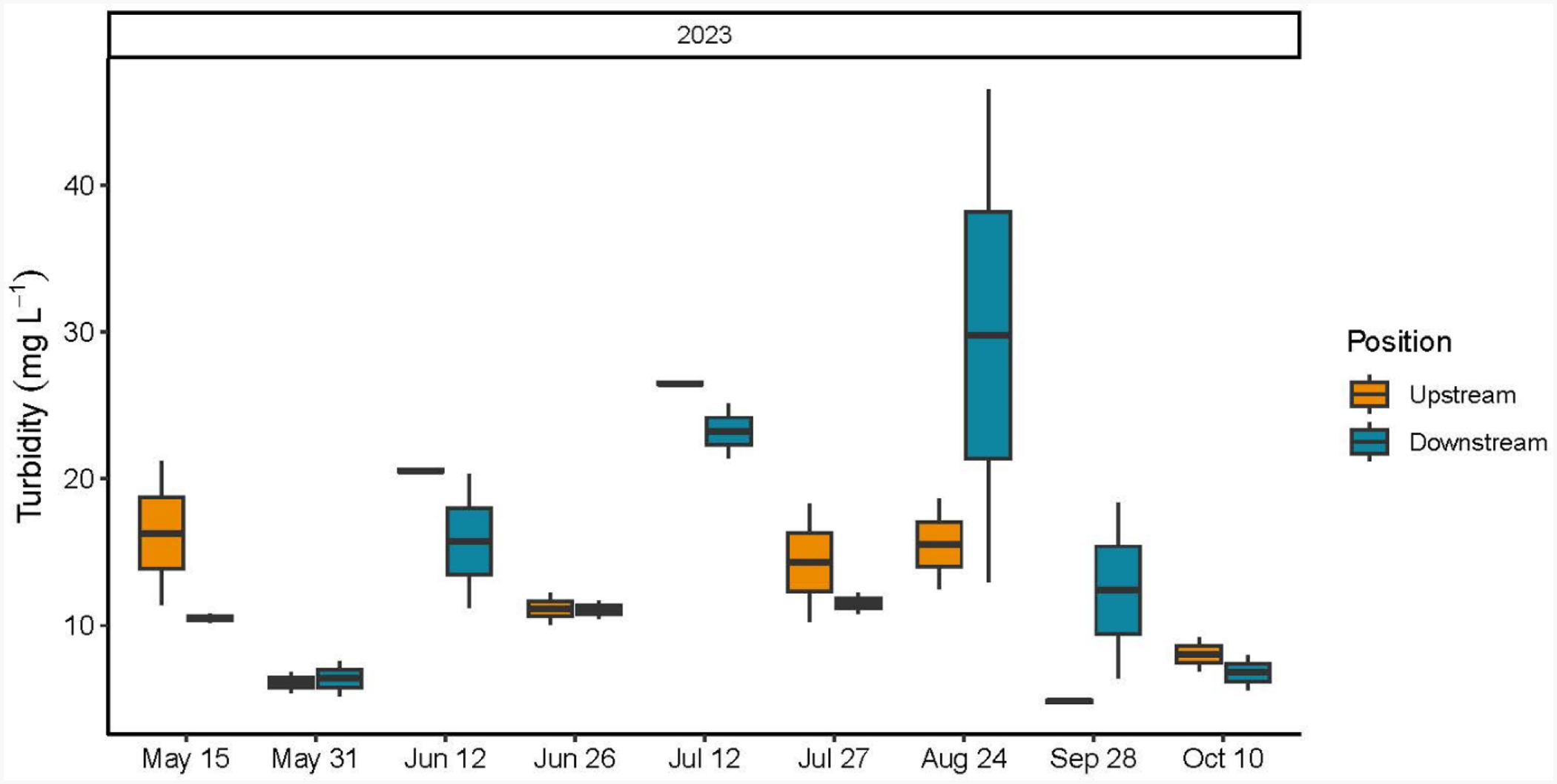
Box and whisker plot including the minimum value, first quartile, median, third quartile, and maximum value of turbidity concentrations (mg L^−1^) from water samples collected upstream and downstream at the start and end of the two-hour sampling window at the Point Judith Pond commercial aquaculture farm between May and October 2023.

**Figure 7.**
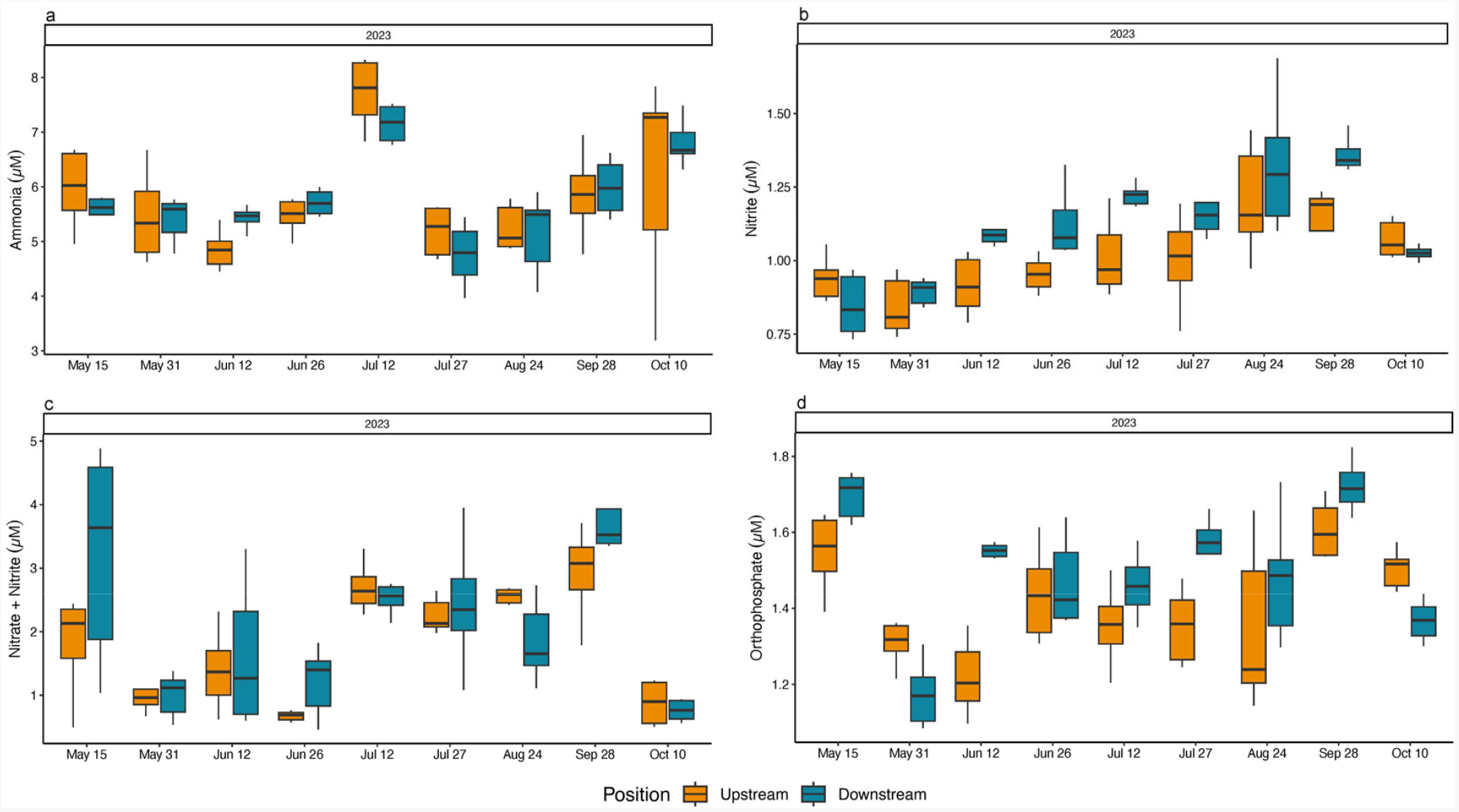
Box and whisker plot including the minimum value, first quartile, median, third quartile, and maximum value of (**a**). ammonia, (**b**). nitrite, (**c**). nitrate plus nitrite, and (**d**). orthophosphate concentrations (μM) respectively, from water samples collected upstream and downstream at the start, middle, and end of the two-hour sampling window at the Point Judith Pond commercial aquaculture farm between May and October 2023.

Individual turbidity values ranged between a minimum value of 4.70 mg L^−1^ to a 10-fold increase of 46.55 mg L^−1^, with no significant interaction between date and position or overall position differences noted (Figure 6, Tables 1, S1 and S2). Upstream average NH_3_^+^ concentration was 6.33 (±3.55) μM, the average downstream value was 5.86 (±1.25) μM (Figure 7a, Table S2), and there were no significant interactions between date and position or overall position differences (Table S1). Upstream average NO_2_^−^ concentration was 1.00 (±0.17) μM and the downstream average NO_2_^−^ value was 1.12 (±0.23) μM (Figure 7b, Table S2). There was a significant overall position effect with downstream values > upstream values (F = 14.57, *p* = 0.0002) (Table S1). The upstream average NO_x_^−^ concentration was 1.75 (±0.89) μM and the average downstream NO_x_^−^ value was 1.99 (±1.21) μM, with no significant interactions or overall position differences (Figure 7c, Tables S1 and S2). The average upstream PO_4_^−3^ value was 1.39 (±0.17) μM and the average downstream value was 1.48 (±0.18) μM. Downstream PO_4_^−3^ concentrations > upstream PO_4_^−3^ concentrations on 12 June and 27 July 2023 (F = 4.48, *p* < 0.0001, date by position interaction) (Figure 7d, Table S1). Spearman rank correlations of Chl *a* and turbidity with the measured biophysical variables (temperature, salinity, DO, nutrients, wind speed and direction, current speed and direction, water depth and Secchi depth) did not produce noteworthy associations across sampling dates in 2023.

One-time measures of seasonal net daily productivity collected upstream, downstream, and within the Point Judith farm in spring (May), summer (July), and autumn (September) 2024 demonstrated a significant interaction between position and season on net productivity (F = 36.99, *p* < 0.0001). At the three sampling positions the net productivity was higher in autumn than the other seasons, and at the downstream position net productivity was higher in summer than the spring. The values ranged between a low of 0.42 (±0.05) g C m^−3^ day^−1^ in spring at upstream to a high value of 6.0 (±0.57) g C m^−3^ day^−1^ in autumn at the oyster farm. The significant season by position interaction showed position differences in the summer and autumn but not during the spring. Specifically, net daily productivity downstream and within farm > upstream net daily productivity in summer, while the net daily productivity in autumn was highest at the within farm position, and the net daily productivity at the upstream position > the downstream position (Table 5).

Generally, the Chl *a* values across sampling locations were lowest in the spring (range = 3.76 ± 0.46 μg L^−1^ downstream to 4.05 ± 0.44 μg L^−1^ within farm), followed by the autumn values (range from 6.82 ± 3.09 μg L^−1^ downstream to 7.34 ± 4.53 μg L^−1^ within farm), with the highest values reported in the summer (range from 8.97 ± 0.79 μg L^−1^ upstream to 11.70 ± 1.30 downstream) (Table 5). The NH_3_^+^ concentrations ranged from 4.80 μM (±0.29) −5.61 μM (±0.61), with no seasonal or positional differences noted. Nitrite concentrations comparisons showed a season by position interaction (F = 4.83, *p* = 0.0080), with the within farm position having NO_2_^−^ concentrations (5.13 ± 1.21 μM) > upstream (2.08 ± 0.58 μM) or downstream (1.34 ± 0.50 μM) positions in spring, additionally the within farm position NO_2_^−^ concentrations in spring (5.12 ± 1.21 μM) > summer (2.15 ± 1.17 μM) and autumn (0.97 ± 0.01 μM). The NO_x_^−^ concentration showed a significant season by position interaction (F = 3.14, *p* = 0.040), specifically, in the spring, NO_x_^−^ concentrations from the within farm (5.70 ± 1.23 μM) > downstream (1.54 ± 0.624 μM). The upstream position exhibited no differences in NO_x_^−^ concentration values by season, while the within farm position had NO_x_^−^ concentrations in spring (5.70 ± 1.23 μM) and summer (5.12 ± 1.22 μM) > NO_x_^−^ concentrations in autumn (1.15 ± 0.30 μM), and the downstream position had NO_x_^−^ concentrations in summer (4.80 ± 0.18 μM) > either spring (1.54 ± 0.62 μM) or autumn (1.21 ± 0.08 μM). Ortho-phosphate concentrations had an overall seasonal difference in concentration values (F = 13.25, *p* = 0.0002), with autumn (1.45 ± 0.10 μM) > spring (1.14 ± 0.15 μM) and summer (1.15 ± 0.10 μM) concentrations (Table 5).

### 3.3. Between Farm Comparisons

A comparison of the measured variables between the two aquaculture farms during the 2023 sampling indicated there were no significant differences between the water temperatures or salinity values; however, DO values were significantly different between farm sites (F = 27.66, *p* < 0.0001) with the Potter Pond DO values greater than Pt Judith values. Unexpectedly, there were no significant differences determined in Chl *a* concentration between the Potter and Pt Judith farm sites. A comparison of turbidity values between the two farm sites demonstrated a significant difference between sites (F = 5.02, *p* = 0.0278) with Potter Pond farm site having higher turbidity values than the Pt Judith Pond farm site. A comparison of the nutrient values reported between farm sites indicated no site difference in NH_3_^+^ concentration; however, there was a significant difference noted in NO_2_^−^ (F = 21.80, *p* < 0.0001), and NO_x_^−^ (F = 15.43, *p* = 0.0001) concentrations with Pt Judith farm significantly > Potter Pond. Ortho-phosphate concentrations were significantly greater at the downstream position at Pt Judith farm as compared to the downstream position at Potter Pond (F = 4.19, *p* = 0.0417).

## 4. Discussion

Investigations of ecosystem benefits and conversely, environmental impacts derived from oyster filtration feeding activities have been considered through laboratory studies [48], via mathematical models [49], on native and restored oyster reefs [22–24], at commercial aquaculture farms [26,27,50,51], and in mesocosms where environmental factors can be controlled and manipulated [29]. In this field study, the findings demonstrate the variable impact of oysters on seston removal (as measured by Chl *a*); however, the results did not conform wholly to the study hypothesis that oyster filtration consistently reduced the Chl *a* concentration across the upstream to downstream gradient at these farm sites during a flooding tide. Based on significant differences in Chl *a* values measured from inside the oyster bags as compared to the upstream Chl *a* concentrations (Potter Pond 2021) and results from the short-term productivity experiment (both ponds 2024), there is evidence to suggest that while oysters are reducing seston there is also localized phytoplankton production, perhaps through nutrient enrichment.

Chlorophyll *a* concentrations at both farm sites ranged between five and eight μg L^−1^ across sampling dates and years (Potter Pond only). This range of Chl *a* values is indicative of mesotrophic conditions characterized as moderately productive and a beneficial location for oyster aquaculture practices. At both farm sites date-specific significant differences were noted in the occurrences of Chl *a* concentrations between the upstream and downstream sampling positions. While it was anticipated that the downstream position would have significantly reduced Chl *a* concentrations, having passed through the oyster farm, this was not observed consistently. Examination of the dates where the downstream Chl *a* concentrations were significantly lower than upstream did not follow a discernible seasonal pattern. Variable Chl *a* concentrations measured at both aquaculture farms point to the complexities of describing the role of oysters in commercial aquaculture operations to regulate nutrient dynamics and improve water quality. Challenges associated with field studies on bivalve filtration at commercial farms, due in part to numerous influential environmental factors such as initial chlorophyll levels, water temperature, resuspension of microphytobenthos during farm operations, microalgae and epibionts associated with the oyster cages, as well as low density oyster farming have been noted [27,50]. Additionally, oyster farm harvesting and restocking practices equate to a change in density and average oyster size throughout the growing season which is difficult to account for in field studies.

As a fresh supply of planktonic organisms are replenished during each flooding tide and with local phytoplankton regeneration, it might be expected there would be top down regulation of this resource by oyster filtration [13,52]; however, depletion of Chl *a* concentrations across the upstream to downstream gradient at aquaculture sites, and at native and transplanted oyster reefs, have not been reported universally and were not established consistently in this study. Measurable seston removal may be dependent upon the individual size and density of oysters [24,27], initial phytoplankton concentrations in the water column [53], other suspension feeding organisms associated with the aquaculture gear (e.g., tunicates and other bivalve species) [54], and environmental factors such as water residence time, wind direction and speed, and current speed [27]. Surprisingly, there was not a correspondingly consistent relationship between the Chl *a* concentrations, turbidity, or nutrient values at the downstream position. The rate of water flow did not have an impact on chlorophyll values, nor was there a relationship with wind direction and speed which has the potential to cause mixing in these shallow water basins, particularly at low tide when water depth was equal or less than 1 m.

In another study, a nearly 20% decrease in Chl *a* concentration was reported downstream at a Chesapeake Bay, (USA) oyster aquaculture farm across multiple sampling events [26]. Counter to this finding, minimal effects from oyster filtration were reported at four other oyster farms in southern Chesapeake Bay, which was attributed to the lowdensity oyster aquaculture [27]. Low Chl *a* concentrations (<10 μg L^−1^) explained the lack of measured differences in upstream and downstream removal rates across the oyster farm in Bournes Pond, MA; however, after an algal bloom produced higher Chl *a* concentrations there was a consistent finding of phytoplankton removal from up- to downstream [53].

The percentage seston depletion by oysters in Potter (average value 2021 = 9.0%, 2022 = 14.3% and 2023 = −14.6%) and Pt Judith Ponds (average value = −5.7%) farms was widely variable and considerably lower than previously measured by other researchers. While positive Chl *a* depletion represents the expected trend of seston removal through filtration along an upstream to downstream gradient, negative values indicate higher Chl *a* concentrations downstream, and an export of Chl *a* (phytoplankton) from the farm system. Studies from oyster reefs in estuarine systems located in the southeast of the USA have generally shown Chl *a* deficits between approximately 10%—and a maximum of 60% [24,25,55]. During the three years of sampling at Potter Pond, Chl *a* concentrations measured from the upstream site (reflecting the incoming water) spiked above 10 μg L^−1^ only four times, typically in September and declining in October. At the Pt Judith Pond farm, Chl *a* concentrations measured from the upstream site rose to 13.92 μg L^−1^ in July and then declined through the later summer and fall. These events may represent sporadic phytoplankton blooms. Generally, low phytoplankton biomass may have an influence on the ability to accurately measure oyster filtration in this study [53]. Independently collected monthly (May through October) water quality data by the University of Rhode Island Watershed Watch program at selected locations in the salt ponds were consistent with the Chl *a* values measured from this study. Two sites in Potter Pond located 239 m and 703 m from the farm had Chl *a* values which ranged from 2.7 ± (1.6) μg L^−1^ to 6.9 (±3.3) μg L^−1^ between 2021 and 2023, and the nearest site located in Point Judith Pond measured 1155 m from the farm had Chl *a* values which ranged from 6.2 (±2.1) μg L^−1^ to 8.1 (±4.8) μg L^−1^ between 2021 and 2022 [56].

Interestingly, the measures of average Chl *a* concentrations drawn from inside the oyster bags in 2021 was >10 μg L^−1^ and double the measured average Chl *a* concentrations reported from the upstream location. Abundant epibionts (e.g., suspension feeders and sponges) attached to the surface of the oyster bags, in between bag cleaning, filter phytoplankton from the water column before the water reaches the oysters growing in the bags. Epibionts are a part of the community; however, unlike an oyster reef, they are not allowed to flourish with the continued cleaning of the aquaculture gear. Despite this pre-filtering, within the bag there is milieu of oyster consumption of incoming phytoplankton, and subsequent deposition of waste products fueling phytoplankton regeneration. This may account for the higher Chl *a* concentration in the oyster bags as compared to the upstream waters and along with tidal water movement may explain the higher Chl *a* concentration at the downstream position. Supporting this suggestion are both the lack of consistent findings for Chl *a* concentration depletion at the two farms, and the seasonal measures of net productivity, which indicate that oyster consumption of phytoplankton and increased deposition waste is enhanced with warming water temperatures. Net productivity was not different between upstream and downstream positions at either farm in the spring but gave way to significantly greater downstream net productivity in the summer suggesting in situ plankton regeneration and horizontal dispersion through the farm with tidal movement. Upstream productivity at Potter Pond in the autumn corresponded to the high Chl values measured upstream. Overall, a weak positive (though non-significant) correlation between Chl *a* concentration and net daily productivity points to the regeneration of phytoplankton associated with the high density of oysters at the farm. At Point Judith, high productivity at the within farm site was not correlated with Chl *a* concentration; however, the occurrences of higher downstream Chl *a* concentrations than upstream suggest either or both, export from the system due to environmental or man-made disturbances to the farm, or regeneration of phytoplankton. Observation of sometimes seasonal variation of phytoplankton productivity upstream, within aquaculture, and downstream positions was an important finding but requires further studies with more frequent measures and improved methodologies (e.g., radiolabelled carbon-14 uptake or oxygen-18 evolution) to account for errors and artifacts associated with the light:dark bottle method.

As water clarity is an ecological indicator of estuarine health, it was anticipated that turbidity would be modulated downstream at the two farm sites because of oyster filtration [57]. Surprisingly, turbidity values did not correlate with changes in the chlorophyll levels at either farm site, nor was there a relationship between turbidity levels and sampling position, except for Potter Pond farm in 2023 when, in general, the downstream value was greater than upstream. This finding suggests phytoplankton regeneration and/or resuspension of benthic microalgae and suspended particulate matter within the farm area moving downstream in the water column influencing turbidity. Similarly, in Chesapeake Bay lower turbidity levels were noted within the farm area than outside the farm for two of the four sites examined [27]. In this study, none of the measured environmental variables thought to be influential were correlated with Chl *a* concentration. Additionally, nutrient levels were not elevated nor were they consistently greater downstream of the farms for any of the years sampled, suggesting they are bound in other dominant components of the system including the microphytobenthos, epiphyte community, and algae associated with the aquaculture structures [58,59]. It is suggested that the direct measures of nutrients from the water column should not be regarded as indicative of eutrophication due to the rapid microbial, algal, and epiphytic uptake in the Rhode Island coastal ponds [40].

## 5. Conclusions

This study examined the effects of two large oyster aquaculture farm sites on a suite of water quality variables in southern RI coastal ponds. Strong and consistent patterns were not identified between Chl *a* concentration, turbidity, nutrients, and the measured environmental factors at the two commercial oyster farms studied during the growing season. Low Chl *a* concentrations may have made it difficult to detect a measurable gradient of phytoplankton filtration from the upstream to the downstream positions. As these are working aquaculture farms normal farm operations such as cleaning algae and epibionts from the oyster bags and harvesting oysters cause resuspension of benthic algae and its movement downstream following water flow may account for the occurrences of higher Chl *a* concentrations at the downstream position as compared to the upstream position. This study also highlights the higher Chl *a* concentrations measured from water collected inside the oyster aquaculture bags, as compared to water from the upstream position at the Potter Pond farm in 2021. This suggests while dense aggregations of oysters in farm bags are feeding on phytoplankton from incoming water; waste as ammonium promotes phytoplankton regeneration. This scenario is repeated on a micro-scale in the hundreds of oyster bags which make up the farm. The productivity study (2024) examined this result on the scale of the farm. The findings point to higher net daily productivity at the downstream (Potter Pond) and downstream and oyster positions (Point Judith) during the summer season, the most biologically active period of growth for the oysters. The complex role of oysters in the aquaculture setting requires further study in the more northern latitudes where seasonal water temperature variations may drive both phytoplankton abundance and the percent of phytoplankton removed by oysters. Natural resource managers might consider all aspects of shellfish aquaculture in northern coastal areas prior to its acceptance and implementation as a method of in-water nutrient remediation.

## Supplementary Material

Supplement1

**Supplementary Materials:** The following supporting information can be downloaded at: https://www.mdpi.com/article/10.3390/coasts6010006/s1, Table S1: Multi-factor GLM analysis of date by sampling position interaction, Upstream (Up) and Downstream (Down), included in the model for both Potter Pond and Pt. Judith Pond oyster aquaculture farms. All variables natural-log transformed prior to analyses, other than Temperature and Salinity. For results with significant date × position interactions, day-specific comparisons done using Bonferroni adjustment; Table S2: Descriptive statistics for measured parameters, including the number of observations (*n*), mean, standard deviation (±SD), minimum (Min), and maximum (Max) values for each parameter, location, year, and sampling position (Upstream = Up, Downstream = Down) from Potter Pond and Pt. Judith Pond oyster aquaculture farms; Table S3: Summary of the mean (*n* = 9) (±SD) chlorophyll *a* (Chl *a*) concentration by date at the Upstream (Up) and Downstream (Down) sampling positions at Potter Pond and Pt. Judith Pond oyster aquaculture farms. Percent Chl *a* removed from the water was calculated as: (Chl *a*) removed = (C1 - C2)/C1 × 100; where C1 = mean Chl *a* upstream value and C2 = mean Chl *a* downstream value.

## Figures and Tables

**Table 1.** Mean, standard deviation (±SD), minimum (Min) and maximum (Max) values for water temperature, salinity, dissolved oxygen (DO), and turbidity from the upstream and downstream sampling positions at Potter Pond (2021–2023) and Pt. Judith Pond (2023). Sampling positions include Upstream (Up) and Downstream (Down) at the oyster farms during a flooding tide.

			Temperature (°C)				Salinity (ppt)				DO (mg L^−1^)				Turbidity (mg L^−1^)			
Site	Year	Position	Mean	SD	Min	Max	Mean	SD	Min	Max	Mean	SD	Min	Max	Mean	SD	Min	Max
Potter	2021	Up	22.4	3.2	16.0	27.5	29.3	0.7	27.8	30.9	11.3	2.7	6.4	15.9	21.8	24.7	6.8	123.2
Potter	2021	Down	24.0	2.5	19.5	27.6	28.8	0.8	27.0	29.8	10.5	2.4	6.2	14.2	21.2	17.4	9.3	84.1
Potter	2022	Up	21.0	4.4	15.1	27.6	31.9	0.8	29.3	33.7	10.1	1.7	7.5	13.2	27.4	27.2	4.3	125.5
Potter	2022	Down	22.6	4.0	15.2	28.3	31.7	1.0	29.8	33.1	9.8	1.9	5.3	13.8	23.6	14.6	6.6	62.4
Potter	2023	Up	21.7	4.8	13.4	30.4	30.2	1.0	28.3	32.5	9.4	1.6	5.6	12.6	13.5	4.1	8.1	25.3
Potter	2023	Down	21.6	4.7	13.8	29.6	29.8	1.1	27.9	31.9	9.8	1.2	7.3	12.3	22.2	19.9	9.1	105.1
Pt. Judith	2023	Up	21.7	3.1	17.5	31.0	30.2	2.2	20.2	32.2	9.0	1.8	5.6	13.0	13.7	7.3	4.7	26.6
Pt. Judith	2023	Down	21.9	3.0	17.5	26.9	30.2	1.8	22.6	32.4	8.7	1.6	5.4	12.9	14.2	9.8	5.2	46.6

**Table 2.** Annual comparisons of measured variables from the Potter Pond oyster aquaculture farm 2021–2023. The analysis was performed using a multi-factor GLM, with date included as a nested factor within year. When there was no interaction the pairwise comparisons of years used a Tukey–Kramer test. When there was a significant year by position interaction the Bonferroni correction was used. Chlorophyll *a*, turbidity, and nutrients were natural-log transformed prior to analysis. There was no transformation for the remaining variables. Sampling positions include Upstream (Up), and Downstream (Down) at the oyster farms during a flooding tide, and N/A not applicable.

Parameter	Year × Position (Up vs. Down) Interaction	Overall Year Difference	Year Difference
Temperature	No	No	N/A
Salinity	No	Yes (F = 25.85, *p* < 0.0001)	2022 > 2021, 2023
Dissolved Oxygen	No	No	N/A
Chlorophyll *a*	No	No	N/A
Turbidity	No	No	N/A
Ammonia	No	Yes (F = 4.36, *p* = 0.0229)	2023 > 2022
Nitrite	No	Yes (F = 5.49, *p* = 0.0100)	2023 > 2021
Nitrate plus Nitrite	No	No	N/A
Orthophosphate	No	No	N/A

**Table 3.** Mean, standard deviation (±SD), minimum (Min) and maximum (Max) values for of Chlorophyll *a* concentrations (μg L^−1^) as determined from the Turner fluorometer for Potter and Pt. Judith Pond by year and sampling position. Sampling positions include Upstream (Up) and Downstream (Down) at the oyster farms during a flooding tide.

				Chlorophyll (μg L^−1^)		
Site	Year	Position	Mean	SD	Min	Max
Potter	2021	Up	5.38	2.38	1.99	12.71
Potter	2021	Down	5.22	3.22	0.86	11.23
Potter	2022	Up	7.91	6.17	1.33	36.97
Potter	2022	Down	5.46	3.28	0.94	17.42
Potter	2023	Up	6.43	3.54	0.01	23.12
Potter	2023	Down	7.35	4.08	2.65	25.00
Pt. Judith	2023	Up	7.01	3.73	2.40	16.46
Pt. Judith	2023	Down	7.30	4.25	3.00	23.67

**Table 4.** Mean, standard deviation (SD), minimum (Min) and maximum (Max) values for ammonia (NH_3_^+^ μM), nitrite (NO_2_^−^μM), nitrate plus nitrite (NO_3_^−^ + NO_2_^−^μM), and orthophosphate (PO4_3_^−^μM) concentrations from Potter Pond (2021–2023) and Pt Judith Pond (2023). Sampling positions include Upstream (Up) and Downstream (Down) at the oyster farms during a flooding tide.

			NH_3_^+^ (μM)				NO_2_^−^ (μM)				NO_3_^−^ + NO_2_^−^ (μM)				PO4_3_^−^ (μM)			
Site	Year	Position	Mean	SD	Min	Max	Mean	SD	Min	Max	Mean	SD	Min	Max	Mean	SD	Min	Max
Potter	2021	Up	5.76	0.79	3.63	7.68	0.70	0.10	0.40	0.99	1.28	0.84	0.58	4.04	1.25	0.36	0.59	2.79
Potter	2021	Down	6.25	1.24	3.64	8.93	0.66	0.11	0.37	0.92	1.14	0.52	0.46	3.21	1.15	0.24	0.68	1.61
Potter	2022	Up	5.10	0.80	3.17	7.48	0.83	0.18	0.55	1.29	1.37	0.86	0.59	4.51	1.24	0.24	0.74	1.85
Potter	2022	Down	5.53	1.12	3.75	8.45	0.84	0.20	0.52	1.61	1.35	0.85	0.45	3.56	1.21	0.26	0.67	1.87
Potter	2023	Up	6.45	2.63	3.87	18.53	0.93	0.27	0.37	1.42	1.34	1.22	0.37	8.83	1.32	0.34	0.54	2.09
Potter	2023	Down	6.34	2.46	3.98	23.42	0.92	0.26	0.42	1.38	1.50	1.11	0.40	5.93	1.24	0.29	0.68	1.87
Pt. Judith	2023	Up	6.33	3.55	3.19	29.67	1.00	0.17	0.64	1.44	1.75	0.89	0.50	3.71	1.39	0.17	1.01	1.71
Pt. Judith	2023	Down	5.86	1.25	3.97	12.21	1.12	0.23	0.72	1.91	1.99	1.21	0.46	4.88	1.48	0.18	1.09	1.82

**Table 5.** Summary table of location, season, sampling position, mean (±SD) Chlorophyll *a* (μg L^−1^), net daily productivity (g C m^−3^ d^−1^), ammonia (NH_3_^+^ μM), nitrite (NO_2_^−^μM), nitrate plus nitrite (NO_3_^−^ + NO_2_^−^μM), and orthophosphate (PO4_3_^−^μM) concentrations derived from the light:dark bottle productivity experiment in 2024. Sampling positions include Upstream (Up), within the oyster farm (Oyster), and Downstream (Down) of the farms during the flooding tide.

Location	Season	Position	Chlorophyll *a* (μg L^−1^)	Net Daily Productivity (g C m^−3^ d^−1^)	NH_3_^+^ (μM)	NO_2_^−^ (μM)	NO^−^ + NO_2_^−^ (μM)	PO4_3_^−^ (μM)
Potter	Spring	Up	6.76 (±0.78)	1.11 (±0.14)	5.35 (±0.22)	2.74 (±0.79)	4.00 (±1.05)	0.95 (±0.09)
Potter	Spring	Oyster	8.17 (±0.53)	1.21 (±0.18)	5.45 (±0.21)	3.69 (±0.66)	5.53 (±0.39)	1.03 (±0.18)
Potter	Spring	Down	9.37 (±1.13)	1.38 (±0.22)	5.06 (±0.49)	3.46 (±1.70)	4.97 (±1.91)	0.99 (±0.07)
Potter	Summer	Up	5.77 (±0.51)	0.53 (±0.09)	8.06 (±1.39)	3.05 (±1.39)	4.67 (±1.54)	1.57 (±0.25)
Potter	Summer	Oyster	6.89 (±0.94)	1.39 (±0.34)	7.07 (±1.20)	1.18 (±0.42)	2.04 (±0.76)	1.12 (±0.20)
Potter	Summer	Down	10.48 (±1.06)	2.14 (±0.29)	5.88 (±0.67)	1.41 (±0.50)	3.05 (±1.76)	1.05 (±0.20)
Potter	Fall	Up	8.48 (±0.57)	1.81 (±0.02)	7.87 (±3.66)	1.20 (±0.09)	2.30 (±1.43)	1.53 (±0.31)
Potter	Fall	Oyster	5.29 (±0.31)	0.97 (±0.22)	7.89 (±0.92)	1.30 (±0.11)	2.35 (±1.28)	1.38 (±0.19)
Potter	Fall	Down	5.61 (±1.75)	0.99 (±0.30)	7.21 (±0.28)	1.23 (±0.08)	2.98 (±1.50)	1.40 (±0.01)
Pt. Judith	Spring	Up	4.20 (±0.35)	0.42 (±0.05)	5.41 (±1.36)	2.08 (±0.58)	2.22 (±0.67)	1.03 (±0.74)
Pt. Judith	Spring	Oyster	4.05 (±0.44)	1.02 (±0.02)	5.61 (±0.61)	5.13 (±1.21)	5.70 (±1.23)	1.18 (±0.19)
Pt. Judith	Spring	Down	3.76 (±0.46)	0.78 (±0.22)	4.79 (±0.29)	1.34 (±0.50)	1.54 (±0.62)	1.22 (±0.18)
Pt. Judith	Summer	Up	8.97 (±0.79)	2.57 (±0.29)	5.27 (±0.40)	1.93 (±0.87)	4.21 (±2.85)	1.15 (±0.10)
Pt. Judith	Summer	Oyster	9.44 (±1.54)	3.28 (±0.40)	5.38 (±1.10)	2.15 (±1.17)	5.12 (±1.22)	1.16 (±0.20)
Pt. Judith	Summer	Down	11.70 (±1.30)	3.65 (±0.46)	5.20 (±0.33)	1.77 (±0.38)	4.80 (±0.18)	1.14 (±0.01)
Pt. Judith	Fall	Up	6.83 (±4.02)	5.35 (±0.31)	5.31 (±0.37)	1.05 (±0.15)	2.10 (±1.42)	1.40 (±0.16)
Pt. Judith	Fall	Oyster	7.33 (±4.53)	6.01 (±0.51)	4.80 (±0.16)	0.97 (±0.01)	1.15 (±0.30)	1.35 (±0.07)
Pt. Judith	Fall	Down	6.82 (±3.09)	3.79 (±0.06)	5.22 (±0.88)	1.12 (±0.16)	1.22 (±0.84)	1.62 (±0.05)

## Data Availability

Research data have been deposited in the EPA SciHub data repository at https://doi.org/10.23719/1532387.
