# Supplementary material for: Limited Benefits of Oyster Aquaculture on Water Clarity in Two Rhode Island Salt Ponds
Source: Coasts (Basel). Author manuscript; Available in PMC 2026 May 5. (PMC13137451; doi:10.3390/coasts6010006)
Supplement: Supplement1 [file NIHMS2154675-supplement-Supplement1.zip › coasts-4074768-supplementary.pdf]

Supplemental Table 1. Multi-factor GLM analysis of date by sampling position interaction, Upstream (Up) and Downstream (Down), included in the model for both Potter Pond and Pt. Judith Pond oyster aquaculture farms. All variables natural-log transformed prior to analyses, other than Temperature and Salinity. For results with significant date x position interactions, day-specific comparisons done using Bonferroni adjustment.

**Parameter**	**Year**	**Location**	**Date × Position Interaction**	**Overall Position Effect**	**Position differences**
Temperature (°C)	2021	Potter	Yes (F=11.07, p<0.0001)	N/A	5/19, 6/1, 6/17, 7/15, 8/10, 8/24, 9/27: Down>Up
Temperature (°C)	2022	Potter	Yes (F=120.92, p<0.0001)	N/A	9/30: Up>Down; 8/3, 9/16: Down>Up
Temperature (°C)	2023	Potter	No	Yes (F=13.68, p=0.0003)	Down>Up
Temperature (°C)	2023	Pt. Judith	No	No	N/A
Salinity	2021	Potter	Yes (F=31.84, p<0.0001)	N/A	5/19, 6/17, 8/10, 9/7: Up>Down; 6/1, 7/15: Down>Up
Salinity	2022	Potter	Yes (F=193.97, p<0.0001)	N/A	6/21, 8/19, 9/19: Up>Down; 9/16: Down>Up
Salinity	2023	Potter	Yes (F=3.60, p=0.0002)	N/A	4/13, 6/8, 7/13, 9/6, 10/5: Up>Down
Salinity	2023	Pt. Judith	Yes (F=2.22, p=0.0299)	N/A	7/27: Down>Up
Dissolved Oxygen (mg L^{-1})	2021	Potter	Yes (F=17.72, p<0.0001)	N/A	5/19, 6/30: Up>Down; 9/7: Down>Up
Dissolved Oxygen (mg L^{-1})	2022	Potter	Yes (F=4.81, p<0.0001)	N/A	9/30: Up>Down;
Dissolved Oxygen (mg L^{-1})	2023	Potter	Yes (F=11.45, p<0.0001)	N/A	8/10: Up>Down; 5/26, 6/8, 10/5: Down>Up
Dissolved Oxygen (mg L^{-1})	2023	Pt. Judith	Yes (F=10.73, p<0.0001)	N/A	7/27: Up>Down; 9/28: Down>Up

Parameter	Year	Location	Date × Position Interaction	Overall Position Effect	Position differences
Chlorophyll *a* (µg L^{-1})	2021	Potter	Yes (F=19.15, p<0.0001)	N/A	6/1, 6/17, 6/30, 7/15, 7/29: Up>Down; 8/24, 9/7: Down>Up
Chlorophyll *a* (µg L^{-1})	2022	Potter	Yes (F=16.21, p<0.0001)	N/A	6/21, 8/3, 9/19, 9/30: Up>Down; 7/20: Down>Up
Chlorophyll *a* (µg L^{-1})	2023	Potter	Yes (F=3.41, p=0.0003)	N/A	8/23: Up>Down; 7/13: Down>Up
Chlorophyll *a* (µg L^{-1})	2023	Pt. Judith	Yes (F=12.96, p<0.0001)	N/A	7/27, 8/24: Up>Down; 5/31, 7/12, 9/28: Down>Up
Turbidity (mg L^{-1})	2021	Potter	No	No	N/A
Turbidity (mg L^{-1})	2022	Potter	No	No	N/A
Turbidity (mg L^{-1})	2023	Potter	No	Yes (F=6.20, p=0.0178)	Down>Up
Turbidity (mg L^{-1})	2023	Pt. Judith	No	No	N/A
Ammonia (µM)	2021	Potter	Yes (F=4.38, p=0.0002)	N/A	7/29: Down>Up
Ammonia (µM)	2022	Potter	Yes (F=12.98, p<0.0001)	N/A	8/3, 9/19, 9/30: Down>Up
Ammonia (µM)	2023	Potter	Yes (F=5.41, p<0.0001)	N/A	8/23: Up>Down; 9/6: Down>Up
Ammonia (µM)	2023	Pt. Judith	No	Yes (F=8.59, p=0.0056)	Down>Up
Nitrite (µM)	2021	Potter	No	No	N/A
Nitrite (µM)	2022	Potter	Yes (F=3.16, p=0.0021)	N/A	9/30: Down>Up
Nitrite (µM)	2023	Potter	No	No	N/A
Nitrite (µM)	2023	Pt. Judith	No	No	N/A
Nitrate + Nitrite (µM)	2021	Potter	No	No	N/A
Nitrate+ Nitrite (µM)	2022	Potter	Yes (F=3.63, p<0.0006)	N/A	10/21: Up>Down; 9/30: Down>Up

Parameter	Year	Location	Date × Position Interaction	Overall Position Effect	Position differences
Nitrate + Nitrite (μM)	2023	Potter	Yes (F=4.08, p=0.0003)	N/A	8/23: Up>Down; 9/6: Down>Up
Nitrate + Nitrite (μM)	2023	Pt. Judith	No	No	N/A
Orthophosphate (μM)	2021	Potter	Yes (F=4.45, p=0.0001)	N/A	9/27: Up>Down; 9/7: Down>Up
Orthophosphate (μM)	2022	Potter	Yes (F=4.76, p<0.0001)	N/A (Int.)	6/7: Up>Down; 9/30: Down>Up
Orthophosphate (μM)	2023	Potter	Yes (F=3.13, p=0.0031)	N/A	None
Orthophosphate (μM)	2023	Pt. Judith	No	No	N/A

Supplemental Table 2. Descriptive statistics for measured parameters, including the number of observations (*n*), mean, standard deviation (±SD), minimum (Min), and maximum (Max) values for each parameter, location, year, and sampling position (Upstream = Up, Downstream = Down) from Potter Pond and Pt. Judith Pond oyster aquaculture farms.

Parameter	Location	Year	Position	N	Mean	±SD	Min	Max
Temperature (°C)	Potter	2021	Up	81	22.41	3.28	15.99	27.46
Temperature (°C)	Potter	2021	Down	72	23.99	2.51	19.54	27.60
Temperature (°C)	Potter	2022	Up	83	21.03	4.37	15.09	27.64
Temperature (°C)	Potter	2022	Down	81	22.60	4.03	15.19	28.33
Temperature (°C)	Potter	2023	Up	103	21.69	4.80	13.39	30.40
Temperature (°C)	Potter	2023	Down	94	21.56	4.66	13.80	29.64
Temperature (°C)	Pt Judith	2023	Up	76	21.68	3.12	17.53	30.99
Temperature (°C)	Pt Judith	2023	Down	75	21.85	2.96	17.51	26.93
Salinity	Potter	2021	Up	81	29.30	0.71	27.80	30.91
Salinity	Potter	2021	Down	72	28.83	0.77	27.03	29.78
Salinity	Potter	2022	Up	83	31.89	0.83	29.26	33.66
Salinity	Potter	2022	Down	81	31.73	1.02	29.78	33.11
Salinity	Potter	2023	Up	99	30.19	1.01	28.27	32.45
Salinity	Potter	2023	Down	93	29.83	1.07	27.90	31.91
Salinity	Pt. Judith	2023	Up	74	30.20	2.16	20.24	32.15
Salinity	Pt. Judith	2023	Down	73	30.23	1.76	22.61	32.35
Dissolved Oxygen (mg L^{-1})	Potter	2021	Up	81	11.29	2.70	6.43	15.94
Dissolved Oxygen (mg L^{-1})	Potter	2021	Down	72	10.53	2.38	6.19	14.27
Dissolved Oxygen (mg L^{-1})	Potter	2022	Up	83	10.10	1.65	7.47	13.19
Dissolved Oxygen (mg L^{-1})	Potter	2022	Down	81	9.80	1.91	5.31	13.82
Dissolved Oxygen (mg L^{-1})	Potter	2023	Up	102	9.42	1.57	5.55	12.57
Dissolved Oxygen (mg L^{-1})	Potter	2023	Down	94	9.78	1.18	7.25	12.31
Dissolved Oxygen (mg L^{-1})	Pt. Judith	2023	Up	76	8.96	1.79	5.58	13.04

Parameter	Location	Year	Position	N	Mean	±SD	Min	Max
Dissolved Oxygen (mg L^{-1})	Pt. Judith	2023	Down	75	8.74	1.63	5.42	12.85
Chlorophyll *a* (µg L^{-1})	Potter	2021	Up	90	5.38	2.38	1.99	12.71
Chlorophyll *a* (µg L^{-1})	Potter	2021	Down	81	5.22	3.22	0.86	11.23
Chlorophyll *a* (µg L^{-1})	Potter	2022	Up	90	7.91	6.17	1.33	36.97
Chlorophyll *a* (µg L^{-1})	Potter	2022	Down	90	5.46	3.28	0.94	17.42
Chlorophyll *a* (µg L^{-1})	Potter	2023	Up	103	6.43	3.54	0.01	23.12
Chlorophyll *a* (µg L^{-1})	Potter	2023	Down	103	7.35	4.08	2.65	25.00
Chlorophyll *a* (µg L^{-1})	Pt. Judith	2023	Up	77	7.01	3.73	2.40	16.46
Chlorophyll *a* (µg L^{-1})	Pt. Judith	2023	Down	77	7.30	4.25	3.00	23.67
Turbidity (mg L^{-1})	Potter	2021	Up	20	21.79	24.71	6.80	123.20
Turbidity (mg L^{-1})	Potter	2021	Down	18	21.23	17.44	9.30	84.09
Turbidity (mg L^{-1})	Potter	2022	Up	19	27.35	27.16	4.29	125.50
Turbidity (mg L^{-1})	Potter	2022	Down	19	23.59	14.58	6.59	62.42
Turbidity (mg L^{-1})	Potter	2023	Up	23	13.51	4.07	8.10	25.30
Turbidity (mg L^{-1})	Potter	2023	Down	24	22.22	19.92	9.10	105.10
Turbidity (mg L^{-1})	Pt. Judith	2023	Up	18	13.71	7.26	4.70	26.60
Turbidity (mg L^{-1})	Pt. Judith	2023	Down	18	14.16	9.83	5.20	46.55
Ammonia (µM)	Potter	2021	Up	53	5.76	0.79	3.63	7.68
Ammonia (µM)	Potter	2021	Down	54	6.25	1.24	3.64	8.93
Ammonia (µM)	Potter	2022	Up	60	5.10	0.80	3.17	7.48
Ammonia (µM)	Potter	2022	Down	60	5.53	1.12	3.75	8.45
Ammonia (µM)	Potter	2023	Up	70	6.45	2.63	3.87	18.53
Ammonia (µM)	Potter	2023	Down	70	6.34	2.46	3.98	23.42
Ammonia (µM)	Pt. Judith	2023	Up	52	6.33	3.55	3.19	29.67
Ammonia (µM)	Pt. Judith	2023	Down	52	5.86	1.25	3.97	12.21
Nitrite (µM)	Potter	2021	Up	53	0.70	0.10	0.40	0.99
Nitrite (µM)	Potter	2021	Down	54	0.66	0.11	0.37	0.92
Nitrite (µM)	Potter	2022	Up	60	0.83	0.18	0.55	1.29

Parameter	Location	Year	Position	N	Mean	±SD	Min	Max
Nitrite (μM)	Potter	2022	Down	60	0.84	0.20	0.52	1.61
Nitrite (μM)	Potter	2023	Up	70	0.93	0.27	0.37	1.42
Nitrite (μM)	Potter	2023	Down	70	0.92	0.26	0.42	1.38
Nitrite (μM)	Pt. Judith	2023	Up	52	1.00	0.17	0.64	1.44
Nitrite (μM)	Pt. Judith	2023	Down	52	1.12	0.23	0.72	1.91
Nitrate + Nitrite (μM)	Potter	2021	Up	53	1.28	0.84	0.58	4.04
Nitrate + Nitrite (μM)	Potter	2021	Down	54	1.14	0.52	0.46	3.21
Nitrate + Nitrite (μM)	Potter	2022	Up	60	1.37	0.86	0.59	4.51
Nitrate + Nitrite (μM)	Potter	2022	Down	60	1.35	0.85	0.45	3.56
Nitrate + Nitrite (μM)	Potter	2023	Up	70	1.34	1.22	0.37	8.83
Nitrate + Nitrite (μM)	Potter	2023	Down	70	1.50	1.11	0.40	5.93
Nitrate + Nitrite (μM)	Pt. Judith	2023	Up	52	1.75	0.89	0.50	3.71
Nitrate + Nitrite (μM)	Pt. Judith	2023	Down	52	1.99	1.21	0.46	4.88
Orthophosphate (μM)	Potter	2021	Up	53	1.25	0.36	0.59	2.79
Orthophosphate (μM)	Potter	2021	Down	54	1.15	0.24	0.68	1.61
Orthophosphate (μM)	Potter	2022	Up	60	1.24	0.24	0.74	1.85
Orthophosphate (μM)	Potter	2022	Down	60	1.21	0.26	0.67	1.87
Orthophosphate (μM)	Potter	2023	Up	70	1.32	0.34	0.54	2.09
Orthophosphate (μM)	Potter	2023	Down	70	1.24	0.29	0.68	1.87
Orthophosphate (μM)	Pt. Judith	2023	Up	52	1.39	0.17	1.01	1.71
Orthophosphate (μM)	Pt. Judith	2023	Down	52	1.48	0.18	1.09	1.82

Supplemental Table 3. Summary of the mean (n = 9) (±SD) chlorophyll *a* (Chl *a*) concentration by date at the Upstream (Up) and Downstream (Down) sampling positions at Potter Pond and Pt. Judith Pond oyster aquaculture farms. Percent Chl *a* removed from the water was calculated as: (Chl *a*) removed = (C1 - C2)/C1 × 100; where C1 = mean Chl *a* upstream value and C2 = mean Chl *a* downstream value.

Date	Location	Mean (±SD) Chl *a* ($\mu g\ L^{-1}$) Up	Mean (±SD) Chl *a* ($\mu g\ L^{-1}$) Down	Percent Chl *a* removed
May 19, 2021	Potter	5.91 (1.47)	6.55 (0.40)	-11
Jun 1, 2021	Potter	8.11 (2.93)	4.98 (1.00)	39
Jun 17, 2021	Potter	3.27 (0.88)	1.59 (0.34)	51
Jun 30, 2021	Potter	4.57 (1.56)	2.93 (049)	36
Jul 15, 2021	Potter	3.27 (0.88)	1.59 (0.34)	51
Jul 29, 2021	Potter	3.23 (0.78)	1.74 (0.42)	46
Aug 10, 2021	Potter	5.02 (0.56)	6.24 (1.28)	-24
Aug 24, 2021	Potter	6.08 (1.45)	8.21(1.21)	-35
Sept 7, 2021	Potter	5.22 (1.67)	7.79 (0.37)	-49
Sept 27, 2021	Potter	9.16 (1.07)	10.35 (0.59)	-13
Jun 7, 2022	Potter	5.20 (1.19)	7.50 (1.39)	-44
Jun 21, 2022	Potter	9.56 (5.36)	2.55 (0.37)	73
Jul 20, 2022	Potter	2.88 (1.22)	5.00 (1.09)	-74
Aug 3, 2022	Potter	11.24 (9.96)	3.57 (1.24)	56
Aug 19, 2022	Potter	7.03 (1.35)	6.59 (1.85)	6
Sept 16, 2022	Potter	11.26 (2.19)	12.17 (3.53)	-8
Sept 19, 2022	Potter	16.21 (9.81)	6.25 (1.05)	61
Sept 30, 2022	Potter	7.95 (2.05)	2.86 (0.47)	64
Oct 21, 2022	Potter	5.31 (0.64)	6.23 (1.70)	-17
Nov 2, 2022	Potter	2.44 (0.78)	1.82 (0.63)	25
April 13, 2023	Potter	3.02 (0.34)	3.27 (0.57)	-8
May 10, 2023	Potter	6.51 (1.24)	9.51 (0.76)	-46
May 26, 2023	Potter	6.34 (0.85)	5.00 (1.14)	21

Jun 8, 2023	Potter	6.23 (1.07)	6.43 (0.54)	-3
Jun 30, 2023	Potter	4.77 (0.88)	6.48 (0.70)	-36
Jul 13, 2023	Potter	3.80 (1.85)	5.59 (0.72)	-47
Jul 25, 2023	Potter	5.15 (1.04)	7.93 (1.81)	-54
Aug 10, 2023	Potter	6.23 (0.70)	5.24 (0.98)	16
Aug 23, 2023	Potter	6.31 (0.84)	3.34 (0.37)	47
Sept 6, 2023	Potter	5.04 (1.20)	6.38 (1.21)	-27
Sept 21, 2023	Potter	15.79 (4.27)	18.20 (2.83)	-15
Oct 5, 2023	Potter	7.83 (1.33)	9.61 (0.75)	-23
May 15, 2023	Pt. Judith	3.47 (0.44)	3.87 (0.60)	-11
May 31, 2023	Pt. Judith	2.92 (0.36)	3.92 (0.20)	-34
Jun 12, 2023	Pt. Judith	5.38 (0.89)	4.85 (1.07)	10
Jun 26, 2023	Pt. Judith	5.33 (0.59)	5.14 (0.55)	4
Jul 12, 2023	Pt. Judith	10.99 (0.67)	16.54 (4.37)	-50
Jul 27, 2023	Pt. Judith	13.92 (2.50)	9.89 (1.16)	29
Aug 24, 2023	Pt. Judith	8.33 (1.90)	6.15 (0.86)	26
Sept 28, 2023	Pt. Judith	3.61 (0.42)	5.88 (0.98)	-9
Oct 10, 2023	Pt. Judith	7.64 (0.33)	8.81 (1.0)	-15
